# Parkinson’s disease in the spinal cord: An exploratory study to establish T2*w, MTR and diffusion-weighted imaging metric values

**DOI:** 10.1162/IMAG.a.1015

**Published:** 2025-11-13

**Authors:** Samuelle St-Onge, Camille Coustaury, Caroline Landelle, Linda Solstrand Dahlberg, Ovidiu Lungu, Julien Doyon, Benjamin De Leener

**Affiliations:** NeuroPoly Lab, Institute of Biomedical Engineering, Polytechnique Montréal, Montréal, QC, Canada; CHU Sainte-Justine Research Centre, Montréal, QC, Canada; McConnell Brain Imaging Centre, Department of Neurology and Neurosurgery, Montreal Neurological Institute, McGill University, Montreal, QC, Canada; Département de psychiatrie et addictologie, Université de Montréal, Montréal, QC, Canada; Computer Engineering and Software Engineering, Polytechnique Montréal, Montréal, QC, Canada

**Keywords:** Parkinson’s disease, spinal cord, diffusion MRI, magnetization transfer ratio, T2*-weighted

## Abstract

Parkinson’s disease (PD) is primarily defined by brain pathology, including dopamine neuron degeneration and α-synuclein aggregation. Emerging evidence suggests that the spinal cord is also affected, with ex-vivo studies reporting abnormal α-synuclein protein aggregation within the spinal cord of PD patients. While advanced imaging techniques, such as diffusion tensor imaging (DTI), neurite orientation dispersion and density imaging (NODDI), T2*-weighted (T2*w) imaging, and the magnetization transfer ratio (MTR) have demonstrated potential for detecting PD-related changes in the brain, their application to uncover spinal cord alterations remains unexplored. This study is the first to investigate MRI-derived microstructural metrics in the spinal cord of PD patients, comparing them to healthy controls. Although this study found limited microstructural or structural differences in the spinal cord between PD patients and healthy controls, these findings are consistent with recent results from PD mouse models and complement an earlier functional MRI study using the same cohort, where significant findings were observed. Our lack of significant structural findings may suggest that functional spinal cord changes are more sensitive markers of Parkinson’s disease progression—particularly in relation to clinical measures such as the Unified Parkinson’s Disease Rating Scale (UPDRS). These results highlight the need for further research to better understand how PD-related alterations in the spinal cord compare to normal aging processes and relate to functional changes.

## 1 Introduction

Parkinson’s disease (PD) is a neurodegenerative disease that is defined by both the degeneration of dopamine-producing neurons inside the substantia nigra (Nyatega et al., 2022) and the abnormal accumulation of α-synuclein inside neurons, forming abnormal protein aggregates commonly referred to as Lewy bodies (Bianco et al., 2002; Muller et al., 2005). While PD has traditionally been linked to brain pathology, markers of the disease have been identified in the spinal cord, notably the presence of abnormal aggregation of α-synuclein proteins inside spinal cord axons (Nardone et al., 2020), which travel through the white matter and form dense terminal networks in the gray matter (Del Tredici & Braak, 2012).

Indeed, post-mortem studies have reported a higher number of α-synuclein aggregates in the spinal cord of PD patients compared to their healthy counterparts (Braak et al., 2007; Del Tredici & Braak, 2012). While cases of α-synuclein aggregates found exclusively in the spinal cord are rare, α-synucleinopathy is commonly observed in the spinal cord when it is also present in the brain. This pattern suggests that, in such cases, PD may affect the midbrain before the spinal cord (Del Tredici & Braak, 2012). There is also extensive evidence indicating that PD may originate in the enteric nervous system (ENS) before progressing to the midbrain (Del Tredici & Braak, 2012, 2016), supporting the distinction between body-first and brain-first subtypes of PD (Del Tredici & Braak, 2012, 2016; Horsager et al., 2020; Leclair-Visonneau et al., 2020). Post-mortem studies further suggest that brain-first PD may begin in the olfactory system or the amygdala (Horsager et al., 2020). Nonetheless, the origin of PD within the body remains under debate, along with the mechanisms through which the disease spreads to the spinal cord.

The work of Bloch et al. (2006) has shown a higher concentration of α-synuclein in the lower brainstem than in the spinal cord, which is consistent with the findings of Del Tredici and Braak (2012). Furthermore, Del Tredici and Braak (2012) identified globules filled with α-synuclein in individuals with advanced PD, as opposed to small varicosities in earlier stages, consistent with findings from Sekigawa et al. (2015), who observed axonal swelling associated to α-synuclein in the spinal cord of transgenic mice. Recently, Combes et al. (2024) have studied structural MRI in the spinal cord of transgenic PD mice. Their results revealed no structural changes in the spinal cord of the PD mouse compared with their non-transgenic counterparts. Functional aspects of PD in the human spinal cord have been explored *in-vivo* by Landelle et al. (2023), using functional magnetic resonance imaging (fMRI), where a decrease of functional connectivity in conjunction with disease progression has been observed in the cervical spinal cord. Nevertheless, *in-vivo* spinal cord studies for PD are limited, and the structural aspects of PD on the human spinal cord remain unexplored, hence the relevance of investigating MRI-derived metrics on the human spinal cord, *in-vivo*.

Diffusion-weighted imaging (DWI) is a promising modality to quantify spinal cord microstructure, as it provides information regarding the diffusion of water molecules that can indicate tissue abnormalities and damage. The diffusion tensor imaging (DTI) model is among the most widely used diffusion MRI models, allowing to measure fractional anisotropy (FA), mean diffusivity (MD), radial diffusivity (RD), and axial diffusivity (AD) (O’Donnell & Westin, 2011). Although a few studies have investigated DTI to quantify gray matter integrity such as in healthy aging (Pfefferbaum et al., 2010), DTI is predominantly used to study the white matter, given its strong anisotropy (Assaf & Pasternak, 2008). Recent work has demonstrated the usefulness of DTI to unveil microstructural tissue damage in PD patients for several regions of the brain, such as the substantia nigra and corpus callosum (Atkinson-Clement et al., 2017; Kotian et al., 2020). Notably, a decrease in FA has been reported with PD progression in the brain (Kotian et al., 2020). Yet, although DTI metrics have demonstrated potential for identifying PD-related microstructural changes at the brain level, to our knowledge, these metrics have not been studied in the spinal cord of PD patients.

Another DWI model that shows promise is the Neurite Orientation Dispersion and Density Index (NODDI) (Zhang et al., 2012), which allows tissue voxels to be compartmentalized into intracellular, extracellular, and cerebrospinal fluid compartments. NODDI metrics include the orientation dispersion index (ODI), the isotropic volume fraction (FISO), and neurite density (FICVF). Previous brain-focused studies have reported reduced values for FICVF and ODI in the substantia nigra pars compacta among individuals with PD (Kamagata, Hatano, & Aoki, 2016; Kamagata, Hatano, Okuzumi, et al., 2016); a phenomenon suspected to be linked to the loss of dopaminergic neurons associated with PD in the brain (Kamagata, Hatano, & Aoki, 2016). Also in the brain, Mitchell et al. (2019) found that ODI increased in white matter while decreasing in gray matter, suggesting possible axonal degeneration or disorganization in white matter, and dendritic thinning in gray matter. However, to our knowledge, no *in vivo* studies have demonstrated neuronal loss related to PD in the spinal cord.

Others have explored T2*-weighted (T2*w), or its reciprocal R2* (1/T2*), as a promising method for identifying PD-related changes in the brain. In their study, Schwarz et al. (2018) noticed a decrease in T2*w signal inside the substantia nigra and nigrosomes in subjects with PD compared to healthy individuals. Since T2*w is sensitive to changes in susceptibility, this decrease has been attributed to iron accumulation, which is well documented in the brain of PD patients (Schwarz et al., 2018; Tambasco et al., 2019; Zeng et al., 2024). Studies suggest that iron deposition is responsible for triggering the activation of immune cells in the brain, which, in turn, release substances that cause inflammation and cause oxidative stress. This process may aggravate the loss of dopaminergic neurons and contribute to disease progression in PD patients (Zeng et al., 2024). Iron accumulation related to PD in the spinal cord has not been reported to date. However, given that the involvement of the spinal cord in PD is still not fully understood, exploring T2*w in the spinal cord of PD patients may reveal subtle changes in tissue susceptibility that were not identified in previous post-mortem studies. Similarly, the magnetization transfer ratio (MTR) has demonstrated relevance in the context of PD in the brain, where studies have noted a decrease in MTR in several brain regions such as the substantia nigra in early PD stages, which is thought to also be linked to iron deposition in the brain (Anik et al., 2007; Tambasco et al., 2003, 2011). Magnetization transfer contrast has also been used in studies of the PD brain to investigate neuromelanin loss in the substantia nigra and locus coeruleus (He et al., 2023; Liu et al., 2020), for its potential as a potential biomarker for PD (Sulzer et al., 2018; Zecca et al., 2006). However, despite the interest in studying T2*w and MTR metrics in the PD brain, their investigation in the spinal cord is yet to be explored. Moreover, it is unclear whether these findings apply to the spinal cord, where dopaminergic neuron loss and iron accumulation have not been reported as potential markers of PD.

The present study thus seeks to address all the above-mentioned knowledge gaps by assessing the potential of quantitative MRI to detect microstructural changes in the spinal cord related to Parkinson’s disease. We aim to establish relationships between PD progression and T2*w, MTR, DTI, and NODDI values in the cervical spinal cord. Since the exact onset of Parkinson’s disease cannot be precisely determined, disease duration was not included in our analyses. Instead, disease progression was assessed using the motor component of the Unified Parkinson’s Disease Rating Scale (UPDRSIII) (Movement Disorder Society Task Force on Rating Scales for Parkinson’s Disease, 2003), which is based on motor assessments done by clinicians. While this score has limitations, being based on interviews and clinical observations that may not fully reflect disease severity, it is widely used in clinical practice to assess PD severity. Therefore, we examined whether MRI-derived metrics correlate with UPDRSIII scores. Additionally, PD stage was further explored by categorizing participants into early, mid, and advanced groups based on their UPDRSIII motor scores. The study also seeks to evaluate whether DTI, NODDI, T2*w, and MTR metrics can be used to differentiate PD patients from their healthy counterparts, which could lead to identifying potential biomarkers for PD in the spinal cord.

## 2 Methods

### 2.1 Data acquisition

**Fig. 1.**
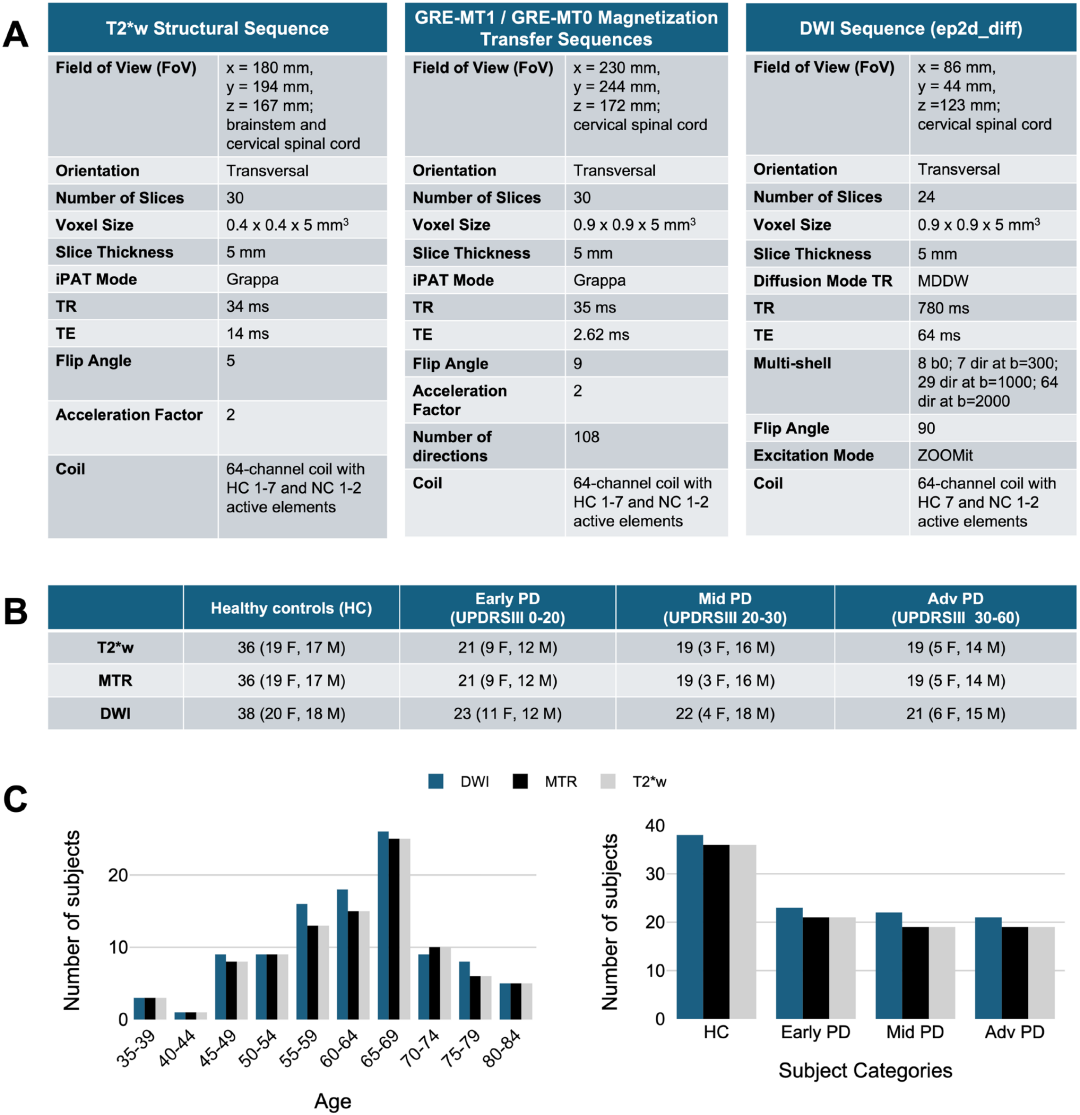
(A) Acquisition parameters for T2*w, MTR, and DWI; (B) Number of participants per category (controls, early PD, mid PD and advanced PD) for each sequence according to their UPDRSIII score. (C) Age and UPDRSIII distributions.

**Fig. 2.**
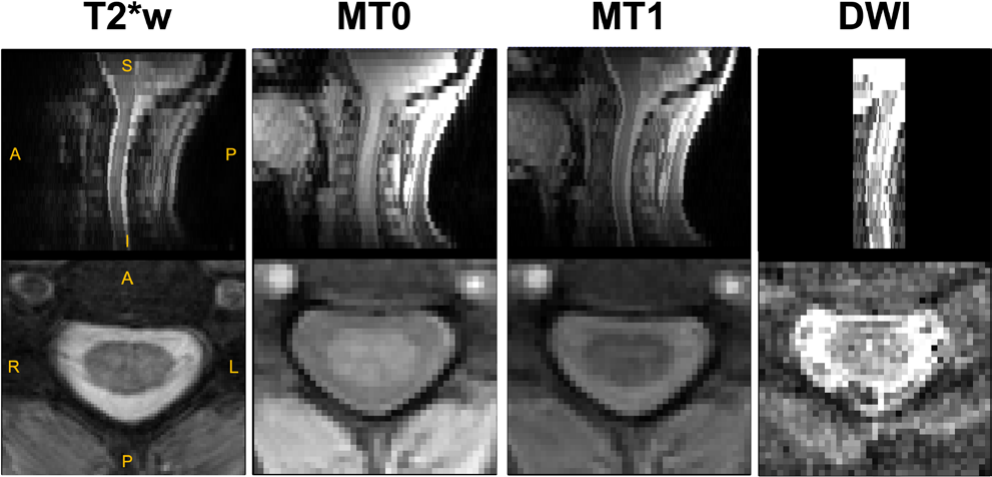
Representative raw data of T2*w, MT0/MT1, and DWI from a single subject.

The study involved the same participants as in Landelle et al. (2023), who have been scanned at the Montreal Neurological Institute-Hospital using a 3 Tesla MRI scanner (Magnetom Prisma, Siemens, Erlangen, Germany). All participants gave their written consent in accordance with the Helsinki Declaration, and the experiment was approved by the local ethics committee (MUCH REB 2019-4626). Participants were required to have no history of other neurological diseases or motor disorders to be included in the study. The acquisition protocol included a T1w, T2*w, magnetization transfer (MT) and a multi-shell diffusion MRI sequence. Participants were positioned supine in the scanner, and the field of view (FOV) was centered on the spinal cord at the C3–C4 disc level and rotated so that the slices were orthogonal to the spinal cord. The detailed acquisition parameters for T2*w, MTR, and DWI are presented in Figure 1. Figure 2 shows examples of raw data acquisitions for a single subject. Because of the limited FOV of the acquisitions, only the cervical spinal cord from spinal levels C2 to C5 was considered for this study.

Participants (N = 106; 65 M, 41 F) included both patients with PD (N = 68; 47 M, 21 F) and matched healthy control subjects (HC) (N = 38; 20 F, 18 M). Individuals with PD underwent clinical interviews and neurological examinations, including an assessment of disease severity with the UPDRS. Using Part III of the UPDRS, which corresponds to the assessment of motor symptoms, participants with PD were further categorized into three group stages: early PD (UPDRSIII 0–20), mid PD (UPDRSIII 20–30), and advanced PD (UPDRSIII 30–60). The participants’ ages ranged from 38 to 83 years old. The mean ages and standard deviations for each group were as follows: overall (N = 106): mean = 64, SD = 10; healthy controls (N = 38): mean = 62, SD = 11; early PD (N = 23): mean = 61, SD = 10; mid PD (N = 22): mean = 64, SD = 7; advanced PD (N = 23): mean = 68, SD = 10. Figure 1B shows the number of participants per category, for each sequence. The age, sex, and UPDRSIII score distribution of the subjects are shown in Figure 1.

### 2.2 Data pre-processing and metric extraction

FA, MD, RD, and AD metrics were computed from the DWI images using DTI from DIPY *via* the Spinal Cord Toolbox (SCT) (De Leener et al., 2017). ODI, FISO and FICVF metrics were computed using the NODDI Matlab Toolbox (UCL Microstructure Imaging Group, 2021).

The current motion-correction algorithm in SCT did not appear to improve image quality on our data. Consequently, subjects with severe motion were excluded from the analysis. For subjects with minimal motion, motion-corrected images were not used, as the correction introduced additional motion in some cases. Therefore, motion correction was not applied; instead, only images with minimal motion that did not affect processing outcomes were retained. It is unclear whether motion in the DWI data was more pronounced in subjects with advanced Parkinson’s disease (PD) in our cohort; however, this remains a potential challenge in studies involving populations with movement disorders.

Using SCT, magnetization transfer (MT) images with and without the MT pulse were co-registered, and magnetization transfer ratio (MTR) maps were computed from the co-registered data. The individual MTR, T2*w, and DWI images underwent initial preprocessing with SCT, which first included spinal cord segmentation using the *sct_propseg* algorithm and manual corrections when necessary. Because of the thick slices of T2*w, MTR, and DWI acquisitions (slice thickness = 5 mm), it was difficult to label the vertebrae on these images directly. For this reason, vertebral labeling was performed on T1w acquisitions (slice thickness = 1.3 mm) and were coregistered to the PAM50 template (De Leener et al., 2018). Then, the generated warping fields were used to initialize the coregistration of T2*w, MTR, DTI, and NODDI images to the PAM50 template. Thus, we generated average group-level metric maps (i.e., average pixel wise intensity) for both HC and PD subjects, at spinal levels C2 to C5 in the PAM50 space.

Using the atlas from the co-registered PAM50 template, we extracted DTI metrics from the white matter and its subregions (dorsal columns, ventral funiculi, and lateral funiculi) to quantify axonal damage, which may be linked to the presence of α-synuclein aggregates in white matter axons, leading to reduced diffusion anisotropy. Similarly, NODDI metrics were extracted from the white matter and its subregions for their sensitivity to axonal integrity and changes in diffusion orientation. Additionally, NODDI metrics were analyzed in the gray matter to detect potential alterations in dendrite orientation and integrity. MTR values were extracted from the white matter and its subregions due to their sensitivity to demyelination, often linked to axonal loss. While MTR is typically assessed in white matter due to its higher myelin content, we also examined it in the gray matter given that post-mortem studies have found dense networks of α-synuclein in spinal cord axon terminals (Del Tredici & Braak, 2012), and the potential for gray matter demyelination or damage. Finally, the WM/GM ratio was calculated from T2*w images using white and gray matter values extracted using the PAM50 atlas. The ratio was chosen because gray matter naturally contains higher iron levels than white matter, and T2*w is particularly sensitive to iron content.

Importantly, all metrics were extracted using the spinal levels (Frostell et al., 2016, SCT v6.1) instead of the vertebral levels to offer a representation that reflects more accurately the functional organization of the spinal cord into different rootlets, thereby enabling more robust comparisons with the findings of Landelle et al. (2023) in functional MRI. The cross-sectional area of the spinal cord was also computed on all individual T2*w images via *sct_process_segmentation* from SCT.

### 2.3 Effect of groups and UPDRSIII score on MRI metrics

We investigated the relationships between PD and MRI-derived metrics (DTI, NODDI, T2*w ratio, and MTR) in the cervical spinal cord by examining how the metrics varied across different groups (controls, early PD, mid PD, and advanced PD). To this end, we performed an analysis of variance (ANOVA) on the mean metric value extracted across spinal levels C2–C5. Age was included as a covariate to control for its potential confounding effects. Post-hoc t-tests were performed on metrics that showed significant results (p < 0.05) to further explore group differences.

To explore the relationship between disease progression and microstructural changes detectable through our metrics, we employed an ordinary least-squares (OLS) regression model to assess the correlations between DTI, NODDI, T2*w, and MTR metrics with UPDRSIII scores, while controlling for age as a potential confounder. For each metric, the OLS analysis was performed for spinal levels C2 to C5 separately. DTI, NODDI, and MTR metrics were studied in the white matter and its subregions (dorsal columns, ventral funiculi, lateral funiculi) to identify potential changes which may be attributed to the accumulation of α-synuclein inside white matter axons. MTR was also studied in the gray matter to detect potential changes related to α-synuclein networks in spinal cord axon terminals. Due to its ability to identify changes in gray matter dendrites, NODDI was also calculated in the gray matter, to investigate whether differences in these metrics could be identified with an increasing UPDRSIII score. Finally, the white matter to gray matter ratio (WM/GM) was studied using T2*w images. To control for the risk of type I errors due to multiple comparisons, the p-values were adjusted using the Benjamini-Hochberg method, also known as the false-discovery rate (FDR) correction. Cross-sectional area (CSA) was also examined using the above-mentioned ANOVA and OLS models to detect potential structural changes in the cervical spinal cord associated with PD.

## 3 Results

### 3.1 Average metric maps

**Fig. 3.**
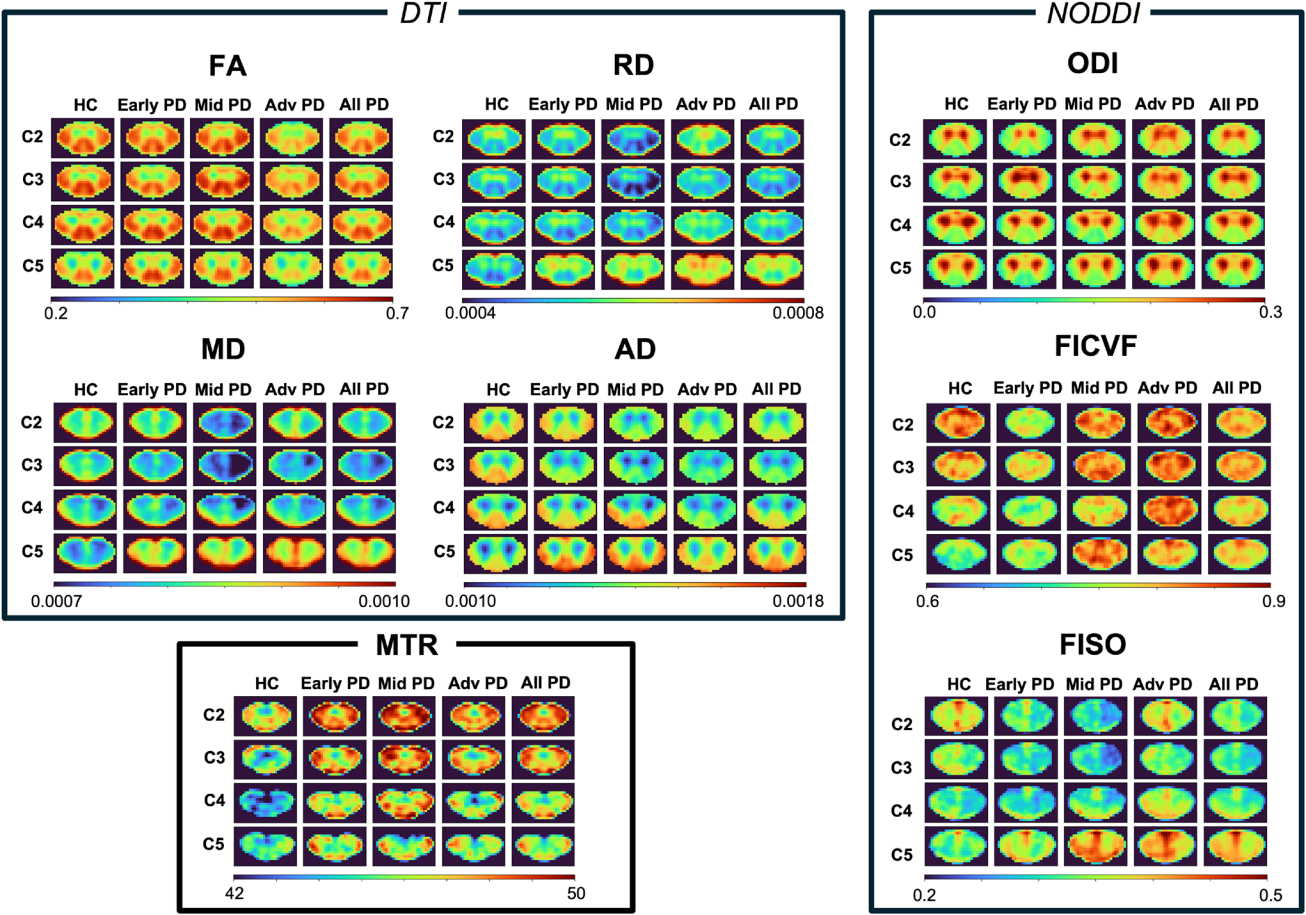
Average spinal cord maps of the DTI, NODDI, and MTR metrics for C2, C3, C4, and C5 spinal levels in healthy control participants (HC), early PD, mid PD, advanced PD and all PD participants combined.

Figure 3 shows the average metric maps for DTI, NODDI, and MTR, obtained by registering the metrics to the PAM50 template and computing the pixel-by-pixel average intensity for each subject category (HC, early PD, mid PD, and advanced PD).

The average metric maps in Figure 3 revealed an increase in ODI and a decrease in FA and AD within the white matter among advanced PD participants compared to their healthy counterparts. However, this difference is not perceived when comparing the HC with all PD subjects combined. For FISO, MD, and RD, we notice the intensity decreases for the early and mid PD groups compared to the HC, and then increases for the advanced PD groups. MTR values are also higher in all PD groups compared to HC. Additionally, a decrease in global intensity is observed in MTR and FA maps as we progress through the more caudal spinal levels, across all subject groups. Interestingly, MD, AD, RD, and FISO values decrease across vertebral levels C2 to C5 in healthy controls, whereas they increase in all PD groups.

### 3.2 Cross-sectional area (CSA) of PD subjects versus healthy controls

**Fig. 4.**
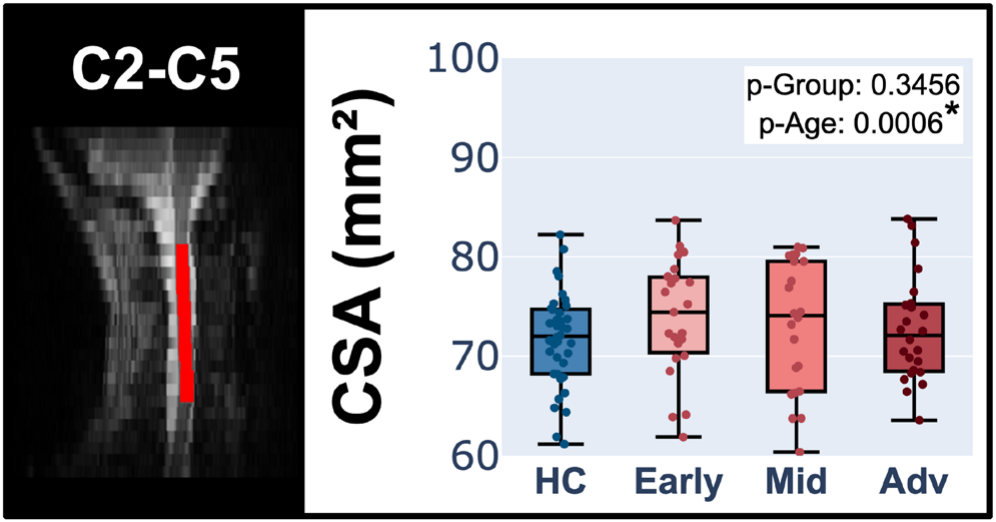
ANOVA for the spinal cord cross-sectional area (CSA) for HC in comparison with PD subjects categorized by their UPDRSIII score (early PD, mid PD and advanced PD), for spinal levels C2 to C5 combined.

Figure 4 presents the results of ANOVA comparing spinal cord cross-sectional area (CSA) across HC, early PD, mid PD, and advanced PD groups, for spinal levels C2 to C5 combined. The results revealed no significant differences in CSA between groups, but a significant effect of age on the CSA (p-Age = 0.0006). Additionally, an OLS analysis revealed no significant correlation between the UPDRSIII score and the CSA (Table 1), but rather a significant correlation of age and CSA at C2, C3, C4, and C5 spinal levels (C2: p-Age = 0.0170; C3: p-Age = 0.0213; C4: p-Age = 0.0170; C5: p-Age = 0.0077). These results suggest that the cervical spinal cord CSA is not affected by PD.

**Table 1.** P-values for UPDRSIII and age variables for the cross-sectional area (CSA) following ordinary least squares (OLS) analysis.

	CSA			
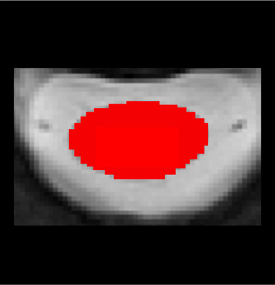	C2	p-UPDRSIII = 0.8743	C4	p-UPDRSIII = 0.6696
		**p-Age** **=** 0.0170*		**p-Age** **=** 0.0170*
	C3	p-UPDRSIII = 0.8111	C5	p-UPDRSIII = 0.8111
		**p-Age** **=** 0.0213*		**p-Age** **=** 0.0077*

The significant p-values (<0.05) are shown in bold with an asterisk (*).

### 3.3 Comparisons across groups (healthy controls, early PD, mid PD and advanced PD)

**Fig. 5.**
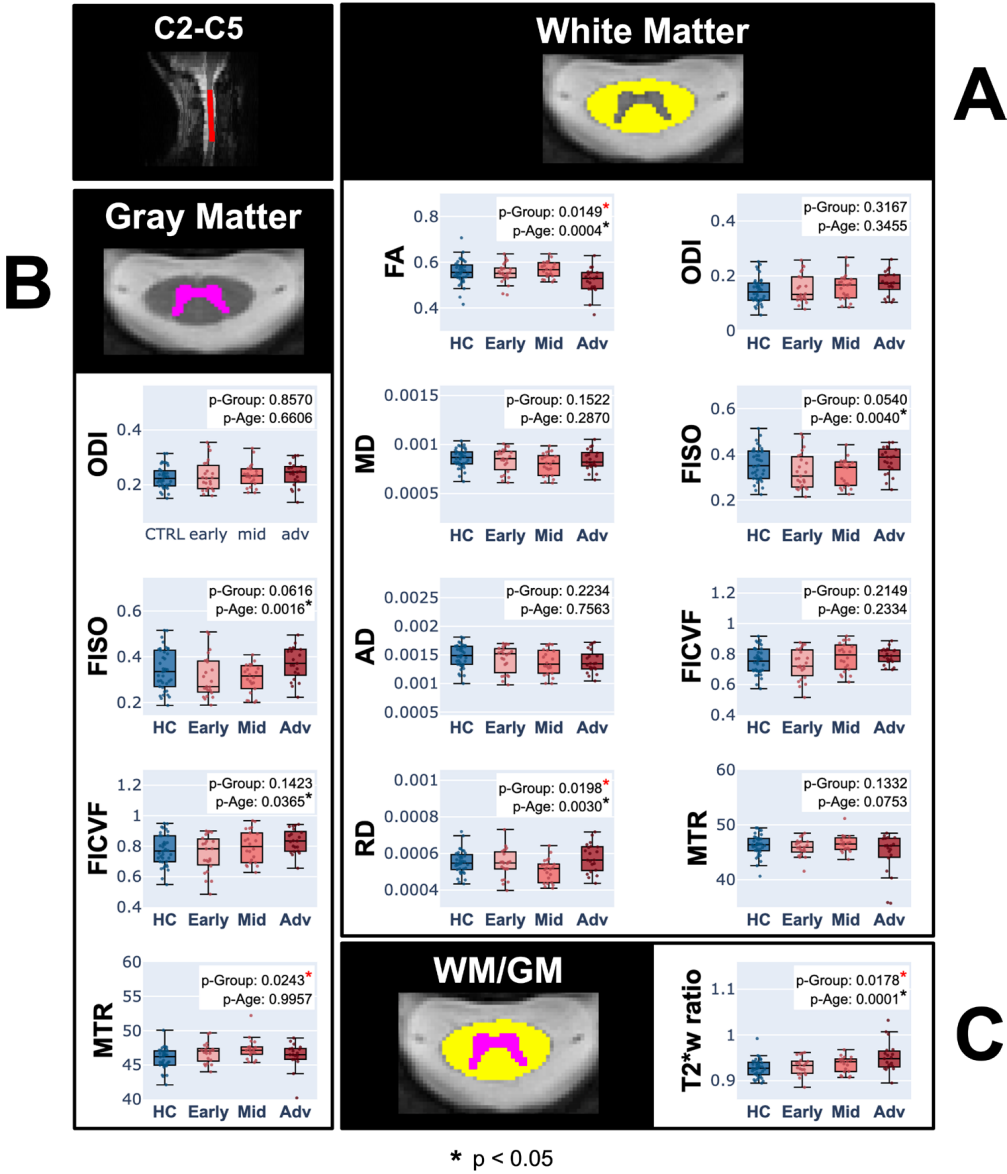
ANOVA for NODDI, DTI, T2*w, and MTR across groups (HC, early PD, mid PD, adv PD) in (A) the white matter, (B) the gray matter, and (C) the white matter to gray matter ratio, for spinal levels C2 to C5 combined. Significant group differences are indicated with a red asterisk (*).

Figure 5 presents the results of an ANOVA comparing DTI, NODDI, MTR, and T2*w metrics across the different groups (HC, early PD, mid PD, and advanced PD), for spinal levels C2 to C5 combined. Here, we notice similar trends to those in Figure 3, where FISO, MD, and RD decrease from HC to mid PD, and then increase again for the advanced PD group. In the white matter, our results showed a significant effect of group on FA (p-Group = 0.0149) and RD (p-Group = 0.0198) metrics. In the gray matter, our data revealed a significant effect of group on MTR (p-Group = 0.0243). Additionally, a significant effect of group was found for the WM/GM ratio in T2*w (p-Group = 0.0178). Post-hoc pairwise t-tests with false discovery rate (FDR) correction were then applied to FA and RD in the WM, to MTR in the GM, and to T2*w in the WM/GM ratio to compare early PD, mid PD, and advanced PD subjects with the HC group (Table 2). Results showed a significant difference between HC and the advanced PD group for FA in the WM (p = 0.0402) and for T2*w in the WM/GM ratio (p = 0.0171), and between HC and mid PD for RD in the WM (p = 0.0386) and MTR in the GM (p = 0.0095).

**Table 2.** FDR-corrected p-values following post-hoc pairwise t-tests between HC – early PD, HC – mid PD and HC – advanced PD.

FA in WM		RD in WM	
Groups	FDR-corrected p-value	Groups	FDR-corrected p-value
HC – early PD	0.7012	HC – early PD	0.6980
HC – mid PD	0.5473	HC – mid PD	0.0386*
HC – adv PD	0.0307*	HC – adv PD	0.3563

MTR in GM		T2*w in WM/GM	
Groups	FDR-corrected p-value	Groups	FDR-corrected p-value
HC – early PD	0.0820	HC – early PD	0.4612
HC – mid PD	0.0095*	HC – mid PD	0.2425
HC – adv PD	0.3932	HC – adv PD	0.0171*

The significant p-values (<0.05) are shown in bold with an asterisk (*).

### 3.4 Correlation analyses with UPDRSIII scores

We employed an ordinary least-squares (OLS) regression model to explore the associations between DTI, NODDI, T2*w, and MTR metrics and UPDRSIII scores, with age included as a covariate to account for its potential confounding effects. Prior to conducting the OLS regression analysis, we assessed the potential collinearity between age and UPDRSIII scores. The Variance Inflation Factor (VIF) found was 1.1, which is well below the commonly used threshold of 10, hence indicating minimal multicollinearity between age and UPDRSIII in our data. This suggests that age and UPDRSIII provide largely independent information, and including both in our model is unlikely to compromise its integrity. Furthermore, with a low Pearson coefficient of r = 0.31, the relationship between UPDRSIII and age appears weak, suggesting that age alone is not a strong predictor of UPDRSIII in this cohort.

Table 3 presents the p-values from the OLS model for the UPDRS-III and age variables, examining DTI, NODDI, and MTR metrics in relation to UPDRS-III score progression across each studied region. FDR correction was applied for each metric, considering all regions and vertebral levels shown in Table 3 (i.e., 16 p-values for MTR and DTI metrics, 20 for NODDI, and 4 for T2*w). P-values in bold text marked with an asterisk (*) indicate statistical significance after applying FDR correction for multiple comparisons. Figures illustrating the regression plots for all metrics with respect to the UPDRSIII score, across each metric and spinal cord subregion, can be found in the Supplementary Material.

**Table 3.** FDR-corrected p-values for UPDRSIII and age predictors for the OLS regression model (metric = UPDRSIII + Age).

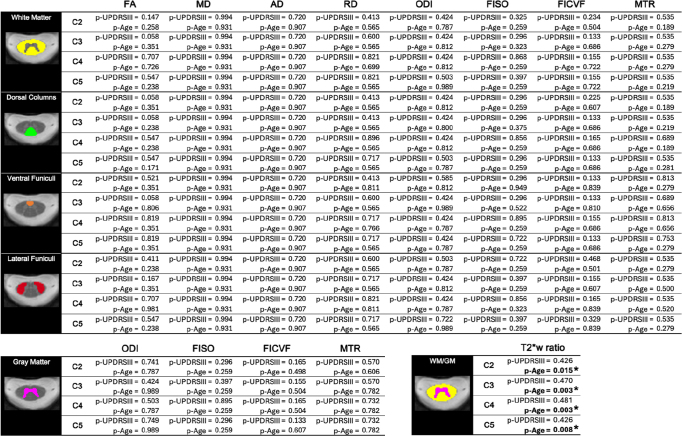

As reported in Table 3, some trends were observed in specific regions, for individual spinal levels and for some of our metrics. Some p-values for the UPDRSIII score were below α = 0.05, but none remained significant after FDR correction. For instance, an increase in ODI, FICVF, and FISO was observed in relation to UPDRSIII across all spinal cord subregions, along with a decrease in FA, which was prominent at the C4 and C5 levels. The OLS regression model revealed an effect of the UPDRSIII on FA for the entire white matter at spinal levels C2 (p-UPDRSIII = 0.046) and C3 (p-UPDRSIII = 0.0138), as well as in some sub-regions of the white matter. Effects of the UPDRSIII score were also observed on other metrics and regions such as RD in the dorsal columns at spinal level C2 (p-UPDRSIII = 0.027), FISO in the ventral funiculi at spinal level C2 (p-UPDRSIII = 0.016), and FICVF in the dorsal columns at spinal level C5 (p-UPDRSIII = 0.034). In the gray matter, the UPDRSIII revealed an effect on the FICVF at C5 (p-UPDRSIII = 0.043). However, when correcting for multiple comparisons, no results were found significant for the UPDRSIII on any of the studied metrics and regions. Age demonstrated a significant correlation with the WM/GM ratio in T2*w for all spinal-level regions (p-Age = 0.007 at C2; p-Age = 0.049 at C3; p-Age = 0.022 at C4; p-Age = 0.022 at C5) after FDR correction.

Regression plots of DTI, NODDI, MTR, and T2*w metrics with respect to age for PD subjects and HC are provided in the Supplementary Material, across all spinal cord subregions and spinal levels. These figures show that the progression of values with age follows a very similar trend in both HC and PD subjects across all metrics and regions. This makes it challenging to differentiate between HC and PD subjects based solely on age-related changes. However, the observed trends, such as an increase in ODI and a decrease in FA, align with findings from previous studies investigating age-related changes in these metrics in healthy individuals (Agosta et al., 2007; Vedantam et al., 2013; Wang et al., 2014).

## 4 Discussion

Based upon our ANOVA analysis, significant differences between HC and some PD groups were identified, notably between HC and the advanced PD group for FA in the WM and for T2*w in the WM/GM ratio, as well as between HC and the mid PD group for RD in the WM and MTR in the GM. In both Figures 3 and 4, we noted a decrease in FISO, MD, and RD in early to mid PD compared to HC, followed by an increase in the advanced PD group. This pattern may indicate different disease mechanisms acting in early to mid stages versus advanced stages of PD, but the underlying cause of this trend remains unclear and requires further investigation. In Figure 3, we observed a decrease in MD, AD, RD, and FISO values when descending from C2 to C5 in healthy controls, whereas these metrics increased in PD subjects. However, when examining HC and PD subjects separately by age for each spinal level (see Appendix B in Supplementary Material), no visual distinction in the metric values is observed from one spinal level to another. Our results also suggest an increase in ODI, FISO and FICVF as well as a decrease in FA with an increasing UPDRSIII score (see Appendix A in Supplementary Material), which may be attributed to the accumulation of α-synuclein, which has been reported to cause axonal swelling (Sekigawa et al., 2015), leading to greater neurite orientation dispersion and reduced diffusion anisotropy. However, since the trends observed in relation to the UPDRSIII score were not significant for any of the metrics after correcting for multiple comparisons (see Table 3), conclusive interpretations cannot be made based on the current study.

Some studies have also investigated the presence of α-synuclein in the spinal cord of individuals without an antemortem diagnosis of PD and without any PD-related symptoms prior to death (Bloch et al., 2006; Buchman et al., 2017; Oinas et al., 2010; Sumikura et al., 2015). Their findings suggest that α-synuclein is relatively common in older adults without a diagnosis of PD, raising the possibility that its presence may serve as a preclinical marker of PD or related disorders, and contributing to a debate over whether such pathology reflects prodromal PD or incidental, non-pathogenic findings. Nonetheless, this could make it even more challenging to distinguish between HC and individuals with a clinical diagnosis of PD, since α-synuclein may also be present in older HC subjects in our cohort without clinical manifestation of the disease.

In our work, we have observed an increase in ODI and decrease in FA in the cervical spinal cord in both HC and PD subjects. These results are consistent with work from other research groups that have explored the effect of age on these DWI metrics in healthy subjects (Agosta et al., 2007). Agosta et al. (2007) have also reported no significant correlation between MD and age, which is also consistent with our findings. Other work with PD in the brain has also reported a negative correlation of FA with age in the substantia nigra (de Oliveira & Pereira, 2021).

Our current study also revealed similar effects of age on our metrics as those observed with the UPDRSIII. In some regions and metrics, such as in the WM/GM in T2*w, our analysis revealed a stronger effect of age than the UPDRSIII on our metrics. This makes it difficult to differentiate between the effects of Parkinson’s disease progression and normal aging. Additionally, while age was included in the analysis, other potential confounding variables that could impact the progression of the disease, such as medication, should be considered in future work.

Previous work have demonstrated relevance for the WM/GM in T2*w as a potential biomarker for spinal cord white matter injury, such as in the case of degenerative cervical myelopathy (Martin et al., 2017). To our knowledge, this study is the first to have explored its potential for PD in the spinal cord. Although we found a significant difference between HC and advanced PD subjects in the T2*w WM/GM ratio, our analysis did not reveal a significant correlation with UPDRSIII scores. Further research will be necessary to establish definitive conclusions regarding the applicability of the WM/GM T2*w ratio in PD.

Finally, in the current study, it was difficult to relate our findings for structural MRI in the spinal cord microstructure with those observed with functional MRI in Landelle et al. (2023) using the same subjects. In their work, Landelle et al. (2023) found a decrease in functional connectivity that correlated with upper limb motor symptoms in between PD patients between C4 and C6 spinal levels. However, our analysis did not reveal any significant microstructural findings that could be associated with the functional changes described in their work. These functional changes may reflect alterations in neural activity or synaptic function, even when the number of neurons or myelination content remains unchanged. For example, disease-related changes in synaptic transmission, glial cell-activity or even spinal cord oxygenation could lead to functional impairments that are not detectable through microstructural MRI. The latter hypothesis is supported by findings from a study in α-synucleinopathy mouse model (Combes et al., 2024), where reduced oxygen saturation in the spinal cord was observed despite the absence of volumetric atrophy. Such results suggest that functional imaging may capture distinct disruptions that microstructural data cannot reveal. Finally, spinal fMRI changes observed in Landelle et al. (2023) may reflect network-level interactions between spinal segments or with the brain, as the authors did not assess local activity but instead reported decreased functional connectivity between consecutive spinal levels involved in upper limb innervation—an interplay not captured by microstructural metrics. Future studies that combine assessments of local activity and microstructural changes could provide deeper insights into the relationship between spinal cord structural and functional changes observed in Parkinson’s disease patients.

Our analysis of spinal cord cross-sectional area (CSA) aligns with the findings of Combes et al. (2024), as we observed no structural changes due to PD. Indeed, our study revealed no significant differences in CSA between Parkinson’s disease (PD) subjects and healthy controls (HC) (see Appendix C in Supplementary Material). Additionally, no significant relationship between the UPDRSIII score and CSA was found. This absence of structural changes could explain the lack of significant correlations between PD and the examined MRI-derived metrics at the microstructural level. Furthermore, the evidence of no structural and microstructural changes within the gray matter may reinforce the findings of Landelle et al. (2023) observed through functional MRI, by suggesting that the effects of PD progression reported in their study were primarily driven by functional changes in the spinal cord rather than anatomical alterations. Nevertheless, subsequent work will be needed to confirm this.

## 5 Conclusion

In summary, this work is the first to have studied DTI, NODDI, MTR, and T2*w metrics in the cervical spinal cord of a population with PD. Although some significant microstructural differences were identified between HC and PD subjects in isolated regions of the spinal cord—notably for FA and RD in the white matter, MTR in the gray matter and T2*w in the WM/GM ratio—no significant changes in microstructural metrics were found in relation to the UPDRSIII score. Our results also suggest that age may have a greater effect on DTI, NODDI, MTR, and T2*w metrics than the UPDRSIII in some regions of the cervical spinal cord. Our lack of significant findings in this current study suggest that the functional changes observed in the spinal cord of the same subjects in Landelle et al. (2023) may be more sensitive markers that correlate with disease progression as assessed by clinical measures, such as UPDRSIII, than the structural measurements. This is consistent with findings from a mixed EEG-MRI study which reported reduced functional connectivity between superior and middle frontal gyrus and paracentral lobule in PD patients relative to healthy controls during an oddball auditory task, while no structural differences were observed in the DTI metrics between the same regions (Droby et al., 2022).

Nonetheless, this exploratory study provides insights into how the cervical spinal cord microstructure is only slightly affected by PD, and how it compares to normal aging processes in the spinal cord. The results are also in line with those of Combes et al. (2024), who did not find any structural changes between PD mice and non-transgenic mice.

## Supplementary Material

Supplementary Material

## Data Availability

The figures in interactive format are available in our preprint via NeuroLibre publication platform: https://github.com/samuellestonge/parkinsons-spinal-cord-MRI-metrics-paper. The extracted DTI, NODDI, MTR, and T2*w metrics for each spinal level (C2 to C5) and spinal cord sub-region are available in .CSV format in our preprint: https://github.com/samuellestonge/parkinsons-spinal-cord-MRI-metrics-paper. The preprint also contains a .CSV file with anonymized participant demographics, such as age, sex, and UPDRSIII scores. The scripts used to perform the statistical analyses as well as to generate the figures are also available in the preprint directory, as well as in the following github repository: https://github.com/samuellestonge/parkinsons-spinal-cord-MRI-metrics-paper. The image processing steps described in this paper were performed using the open-source Spinal Cord Toolbox (SCT), with full details available in the SCT documentation: https://spinalcordtoolbox.com/.
