# Supplementary material for: Parkinson’s disease in the spinal cord: An exploratory study to establish T2*w, MTR and diffusion-weighted imaging metric values
Source: Imaging Neurosci (Camb). 2025 Nov 13;3:IMAG.a.1015. doi: 10.1162/IMAG.a.1015 (PMC12616152; doi:10.1162/IMAG.a.1015)
Supplement: Supplementary Material [file IMAG.a.1015_supp.pdf]

Appendix A: Correlations of DTI, NODDI, MTR and T2*w ratio with the UPDRSIII score

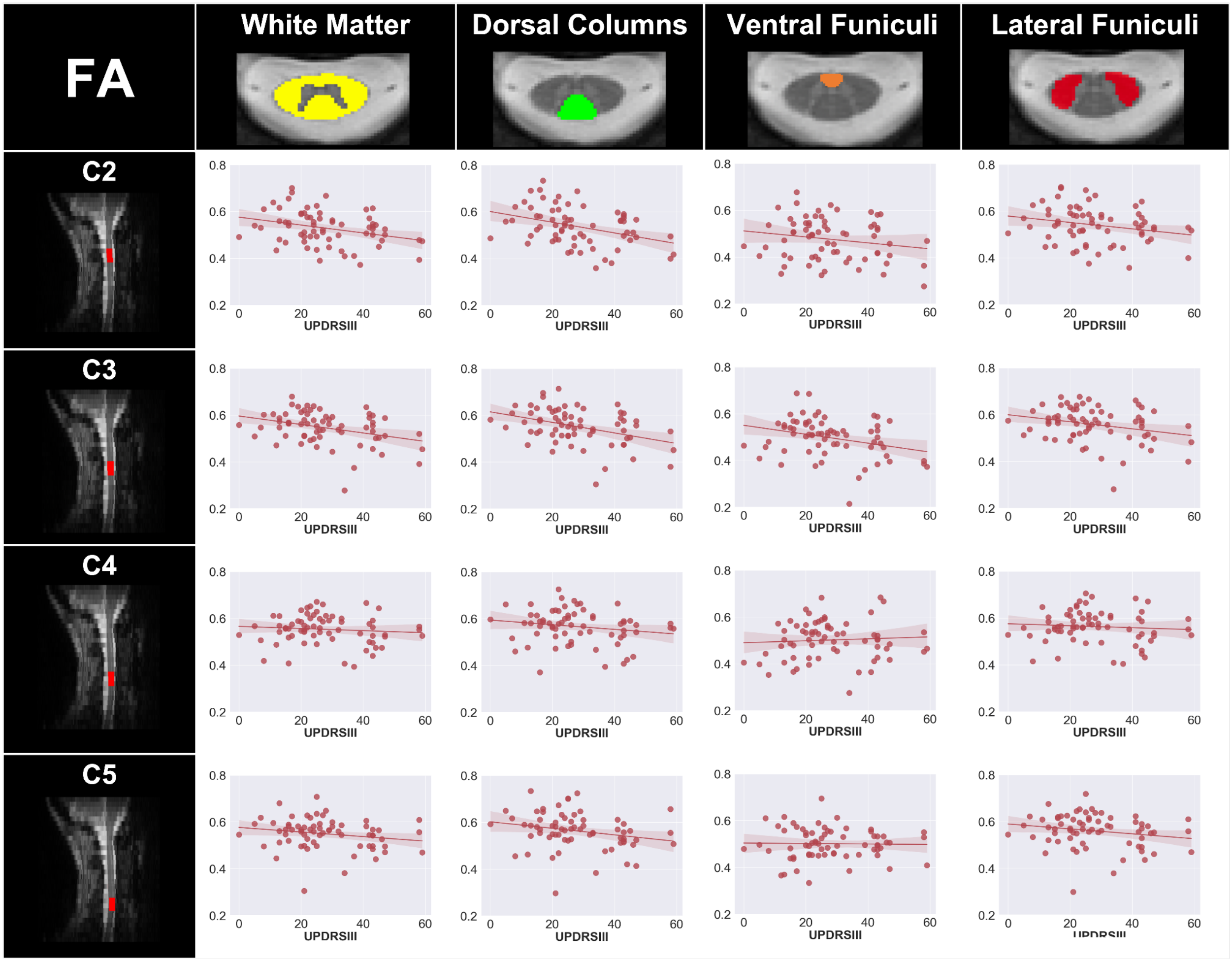

Figure I-1. FA values in PD subjects relative to the UPDRSIII score across spinal levels C2 to C5, for the entire white matter and subregions: dorsal columns, ventral funiculi, and lateral funiculi

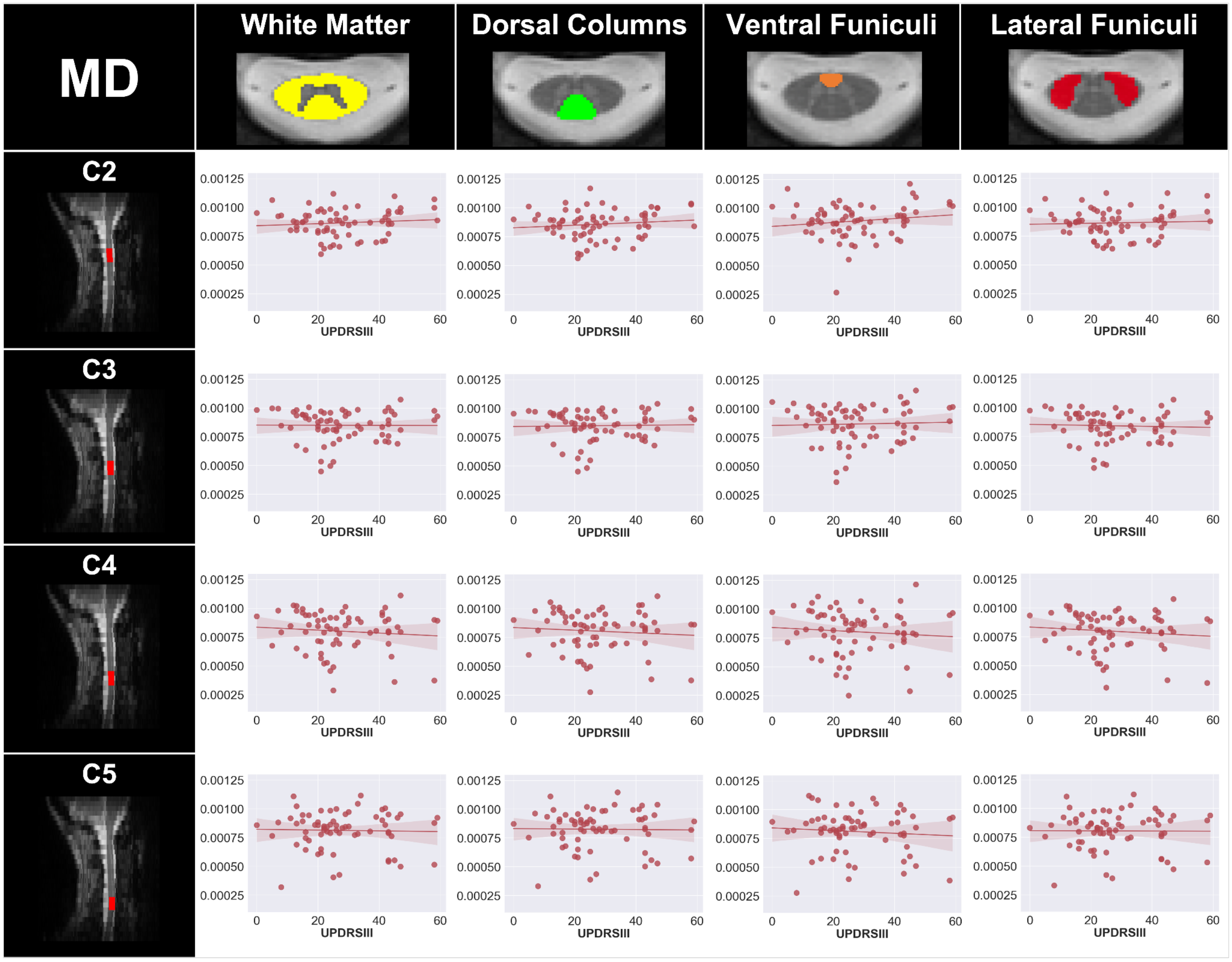

Figure I-2. MD values in PD subjects relative to the UPDRSIII score across spinal levels C2 to C5, for the entire white matter and subregions: dorsal columns, ventral funiculi, and lateral funiculi

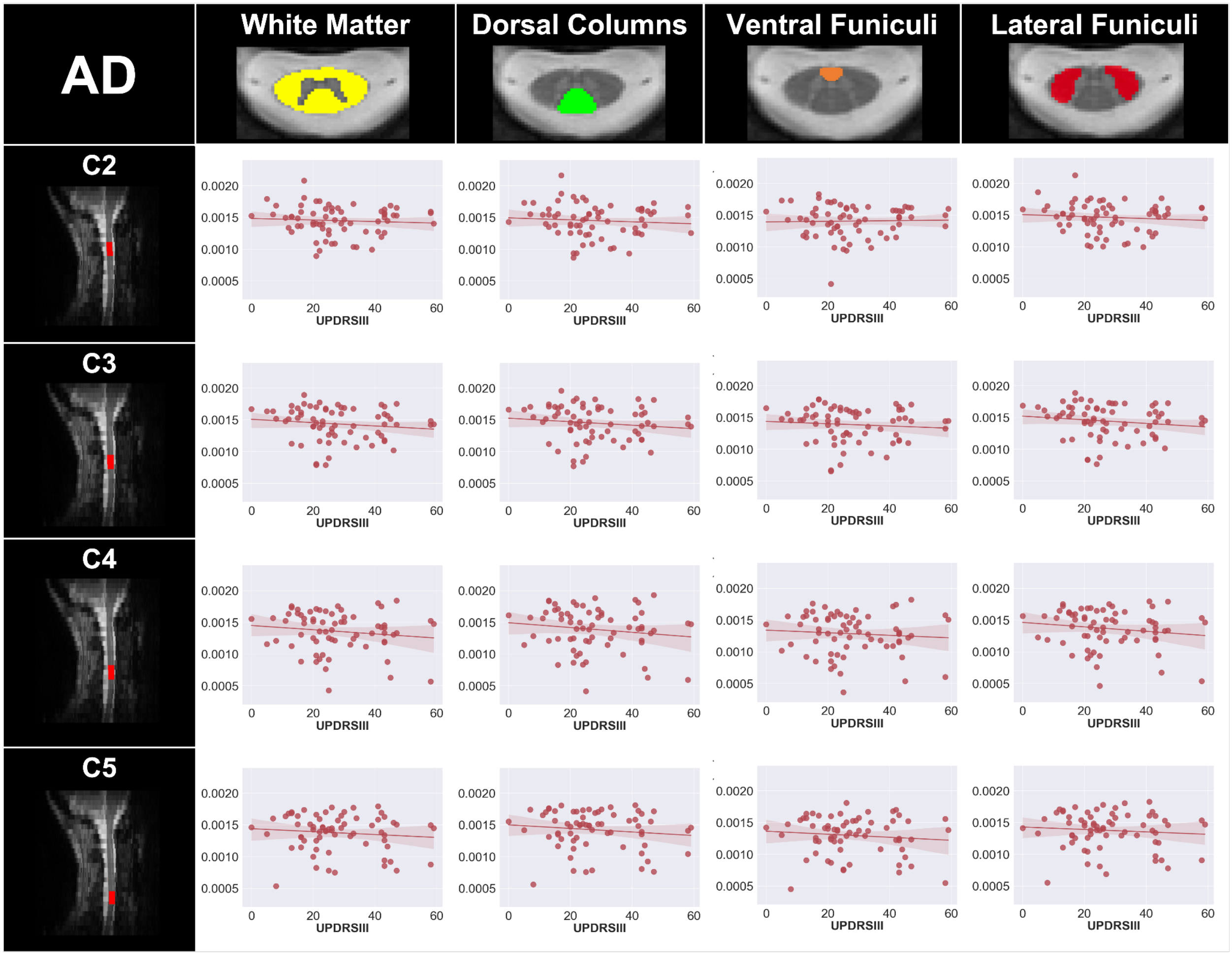

Figure I-3. AD values in PD subjects relative to the UPDRSIII score across spinal levels C2 to C5, for the entire white matter and subregions: dorsal columns, ventral funiculi, and lateral funiculi

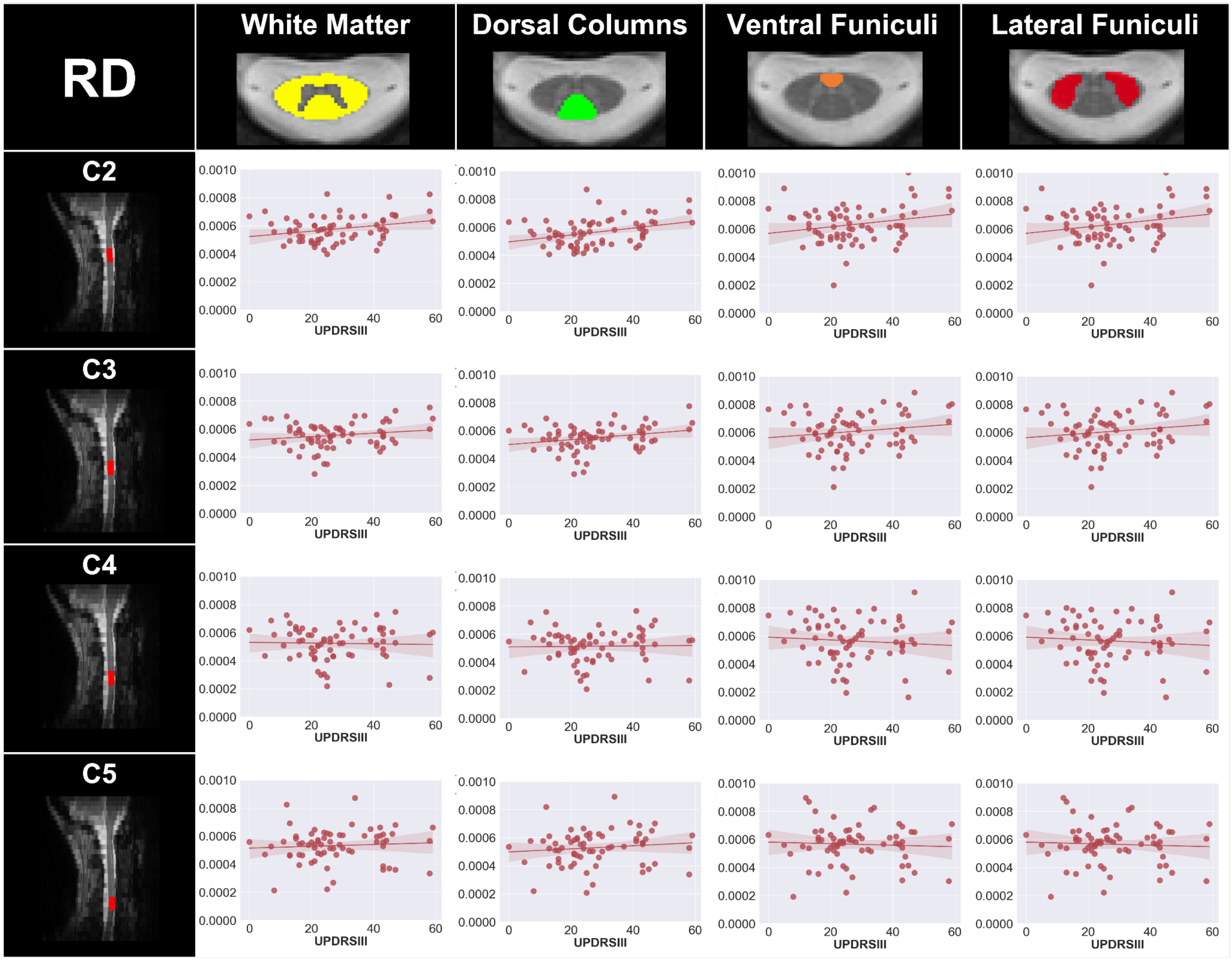

Figure I-4. RD values in PD subjects relative to the UPDRSIII score across spinal levels C2 to C5, for the entire white matter and subregions: dorsal columns, ventral funiculi, and lateral funiculi

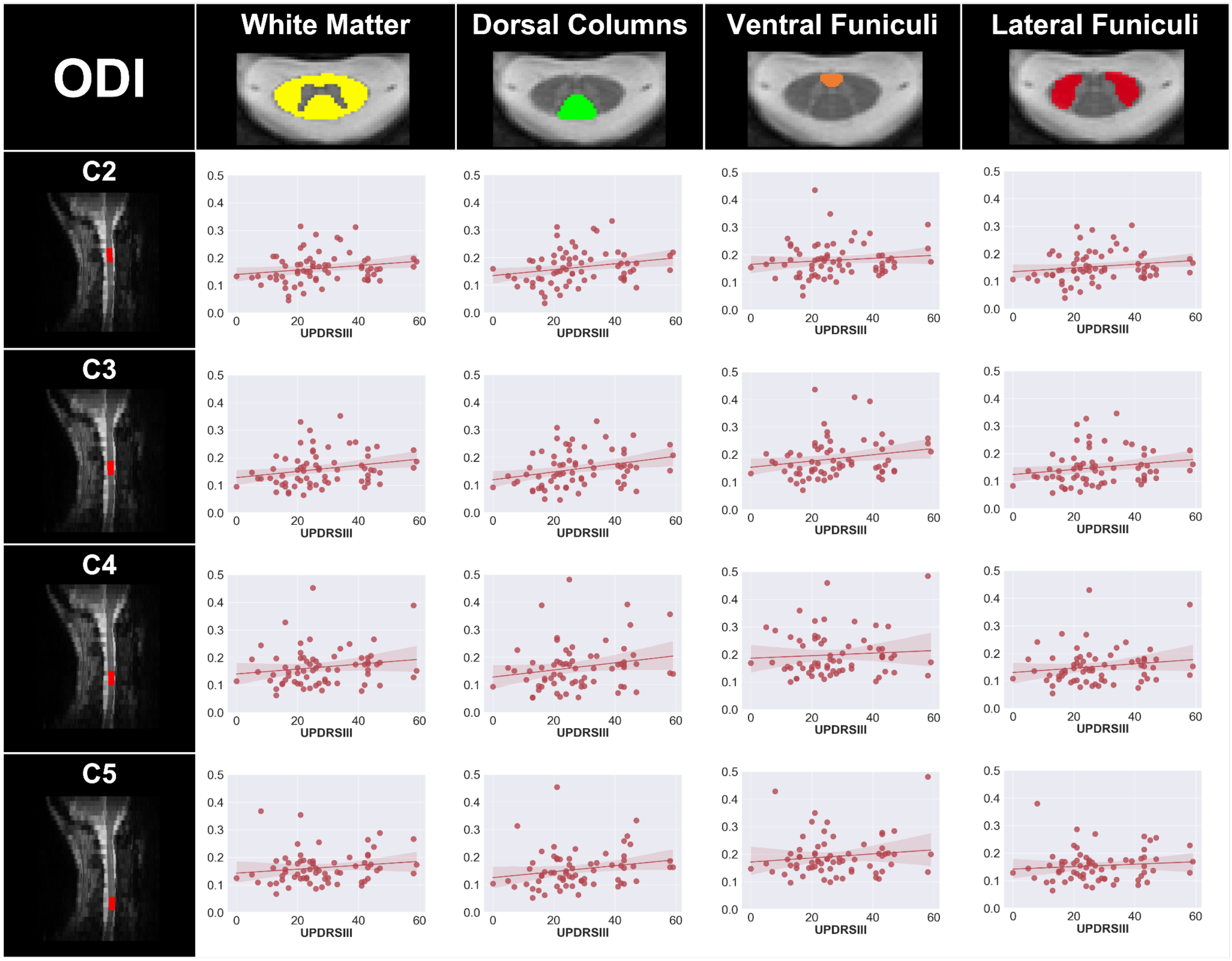

Figure I-5. ODI values in PD subjects relative to the UPDRSIII score across spinal levels C2 to C5, for the entire white matter and subregions: dorsal columns, ventral funiculi, and lateral funiculi

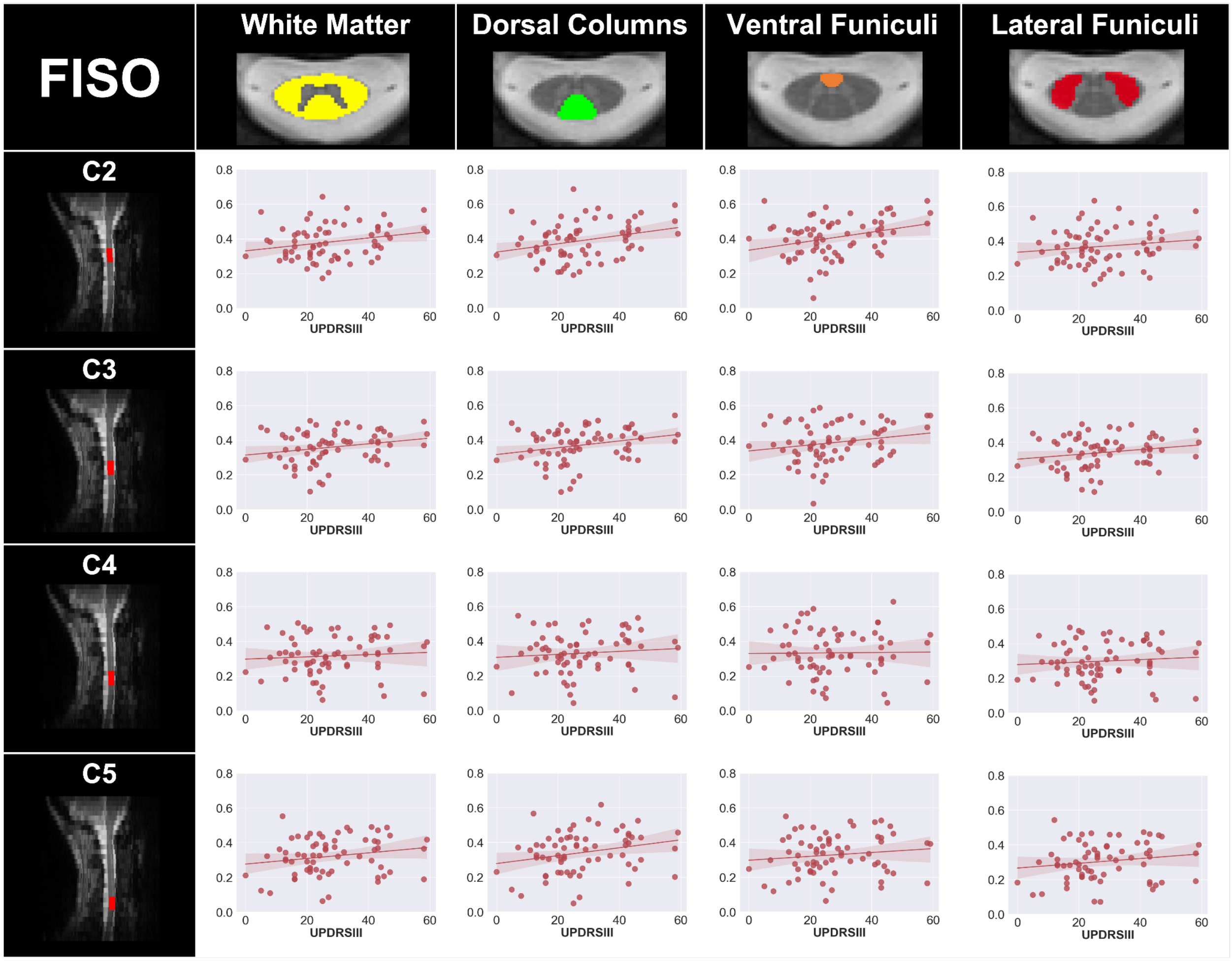

Figure I-6. FISO values in PD subjects relative to the UPDRSIII score across spinal levels C2 to C5, for the entire white matter and subregions: dorsal columns, ventral funiculi, and lateral funiculi

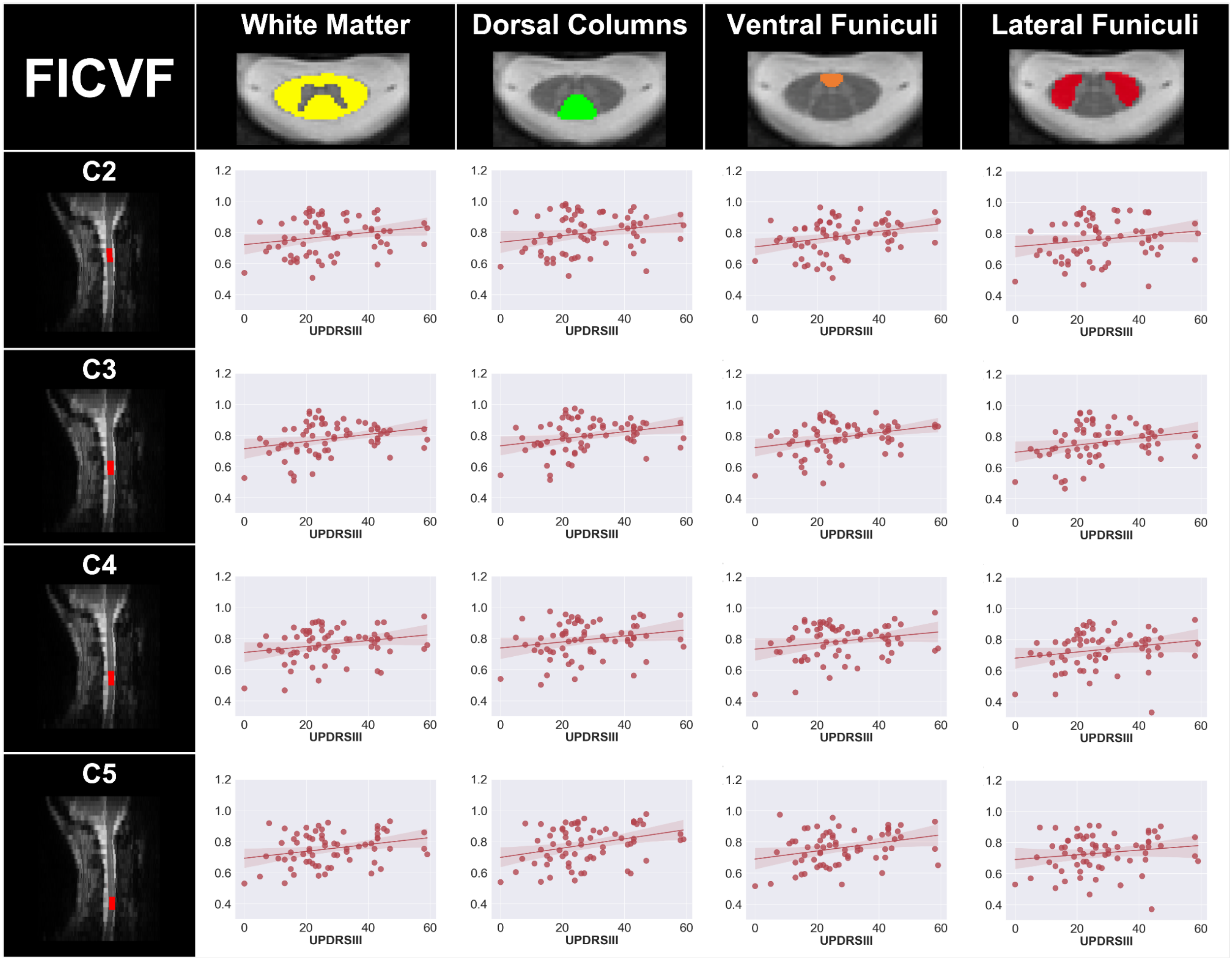

Figure I-7. FICVF values in PD subjects relative to the UPDRSIII score across spinal levels C2 to C5, for the entire white matter and subregions: dorsal columns, ventral funiculi, and lateral funiculi

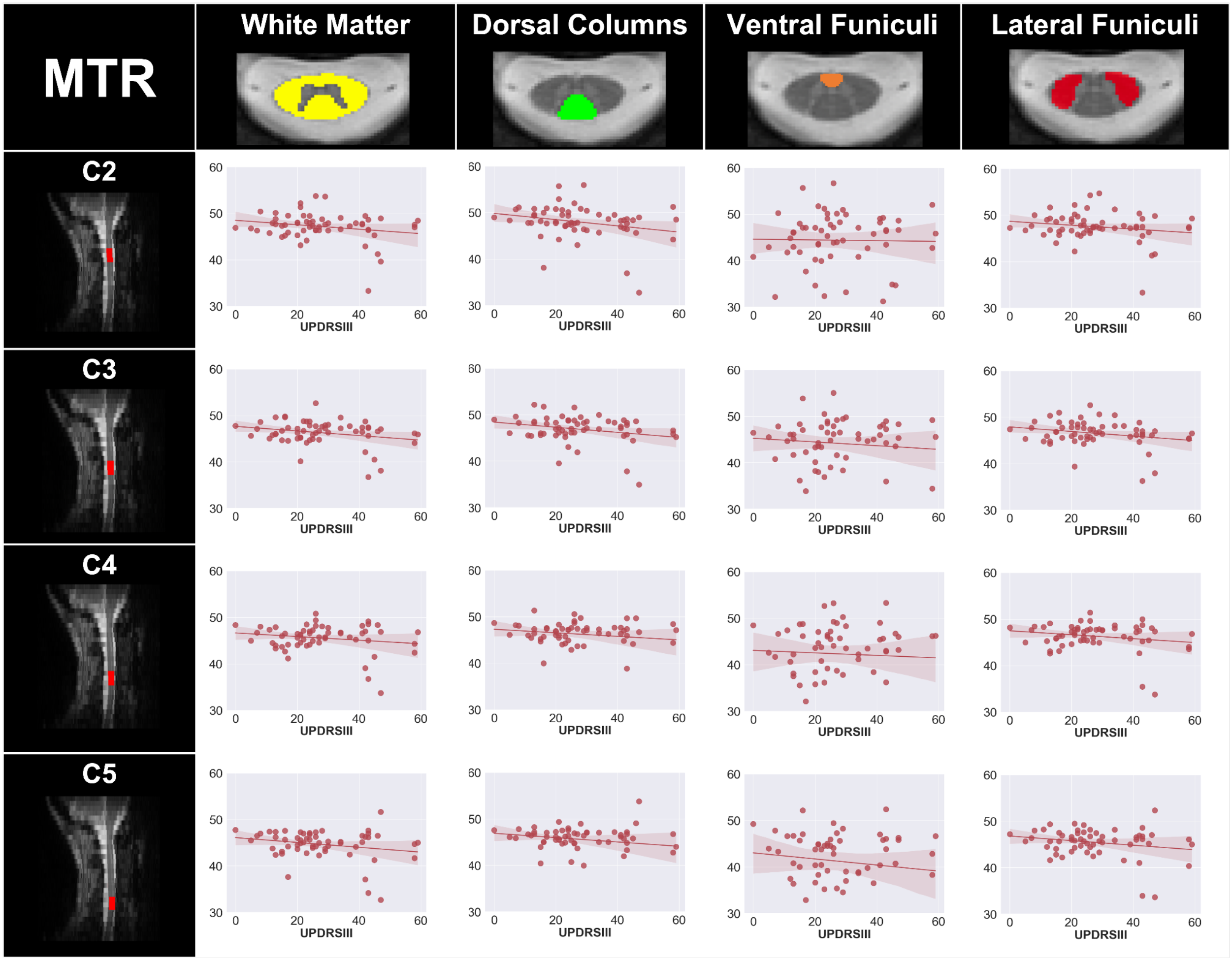

Figure I-8. MTR values in PD subjects relative to the UPDRSIII score across spinal levels C2 to C5, for the entire white matter and subregions: dorsal columns, ventral funiculi, and lateral funiculi

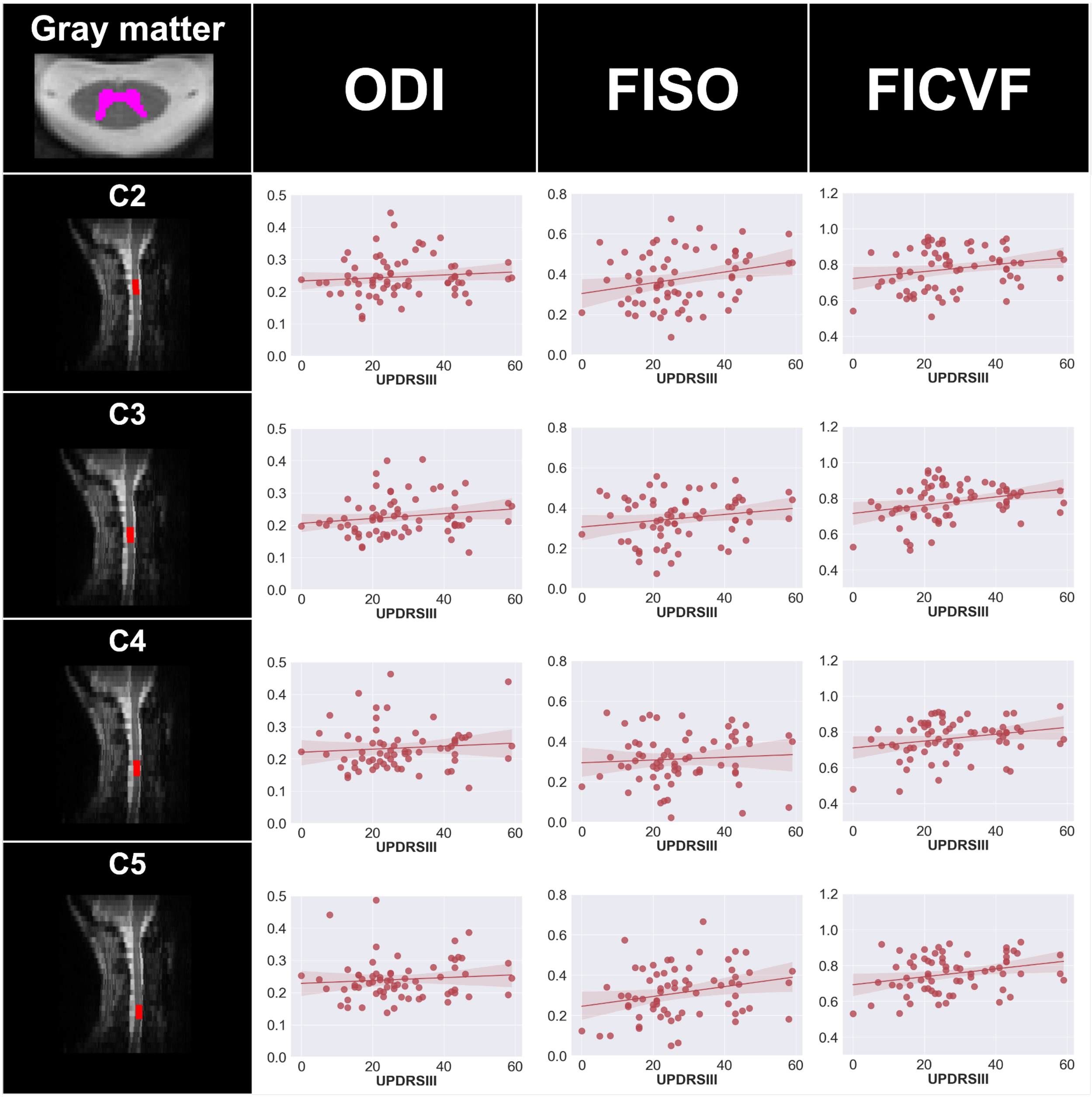

Figure I-9. NODDI (ODI, FISO and FICVF) values in PD subjects with relative to the UPDRSIII score across spinal levels C2 to C5, for the entire gray matter.

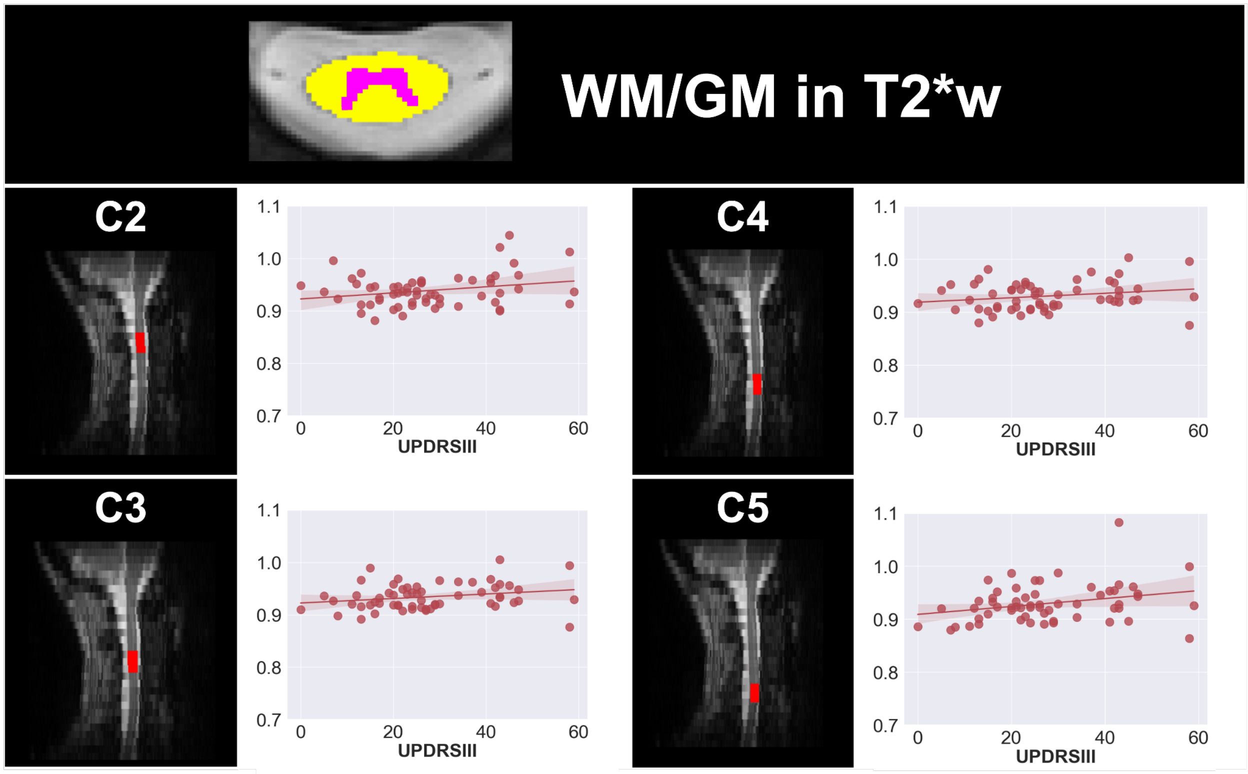

Figure I-10. WM/GM ratio in T2*w in PD subjects relative to the UPDRSIII score across spinal levels C2 to C5.

Appendix B: Correlations of DTI, NODDI, MTR and T2*w ratio with age

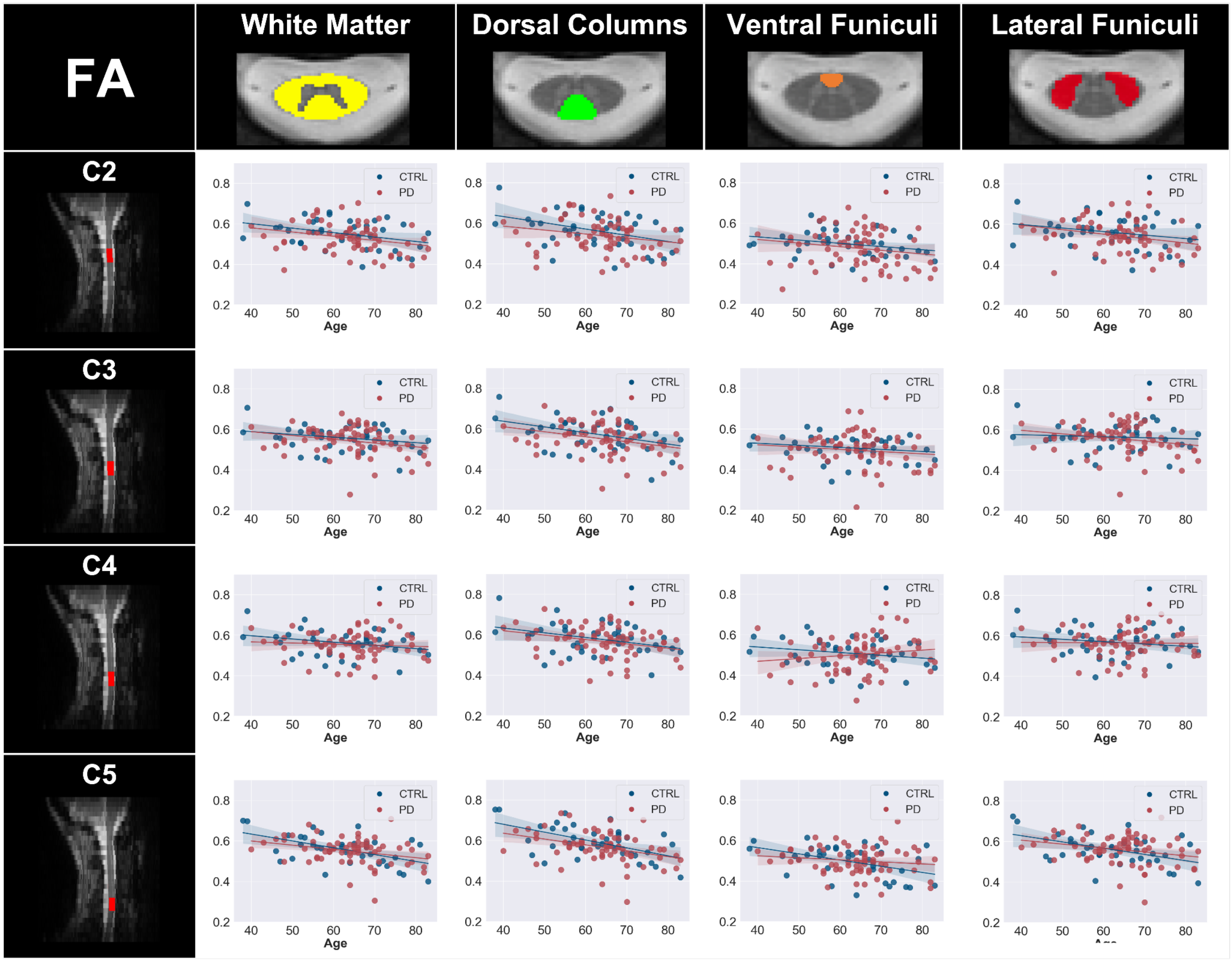

Figure II-1. FA values with respect to age for healthy controls (CTRL) and PD subjects across spinal levels C2 to C5, for the entire white matter and subregions: dorsal columns, ventral funiculi, and lateral funiculi.

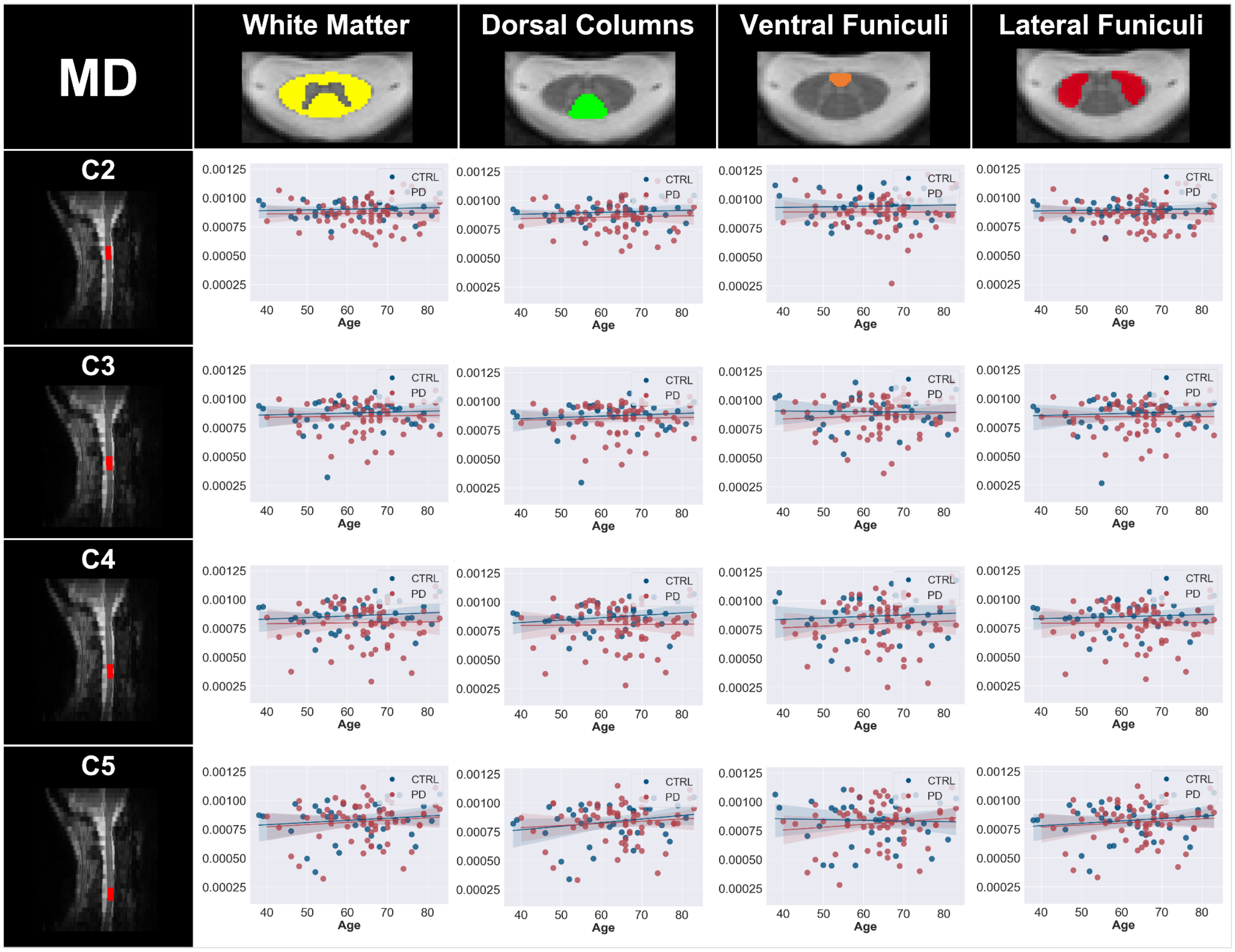

Figure II-2. MD values with respect to age for healthy controls (CTRL) and PD subjects across spinal levels C2 to C5, for the entire white matter and subregions: dorsal columns, ventral funiculi, and lateral funiculi.

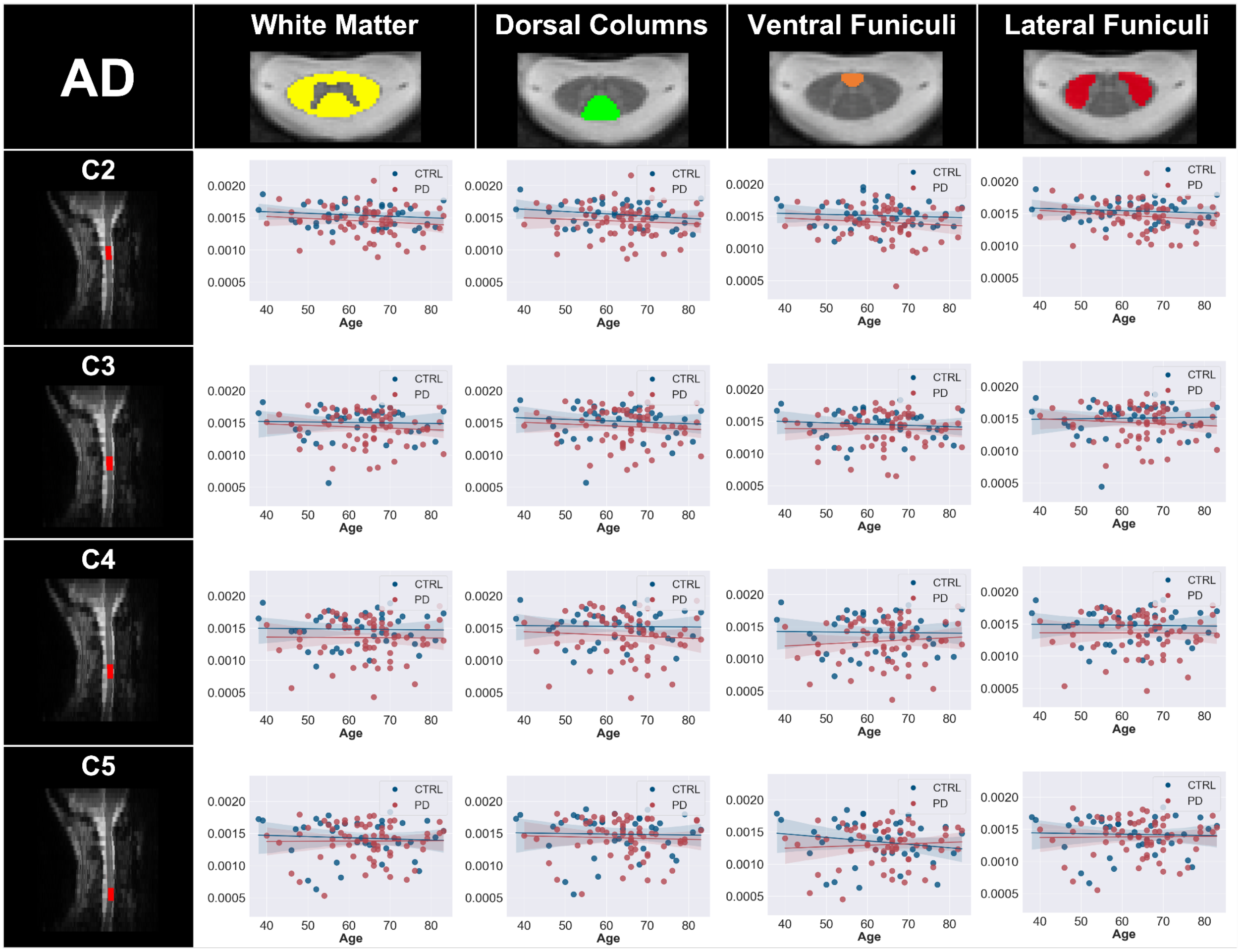

Figure II-3. AD values with respect to age for healthy controls (CTRL) and PD subjects across spinal levels C2 to C5, for the entire white matter and subregions: dorsal columns, ventral funiculi, and lateral funiculi.

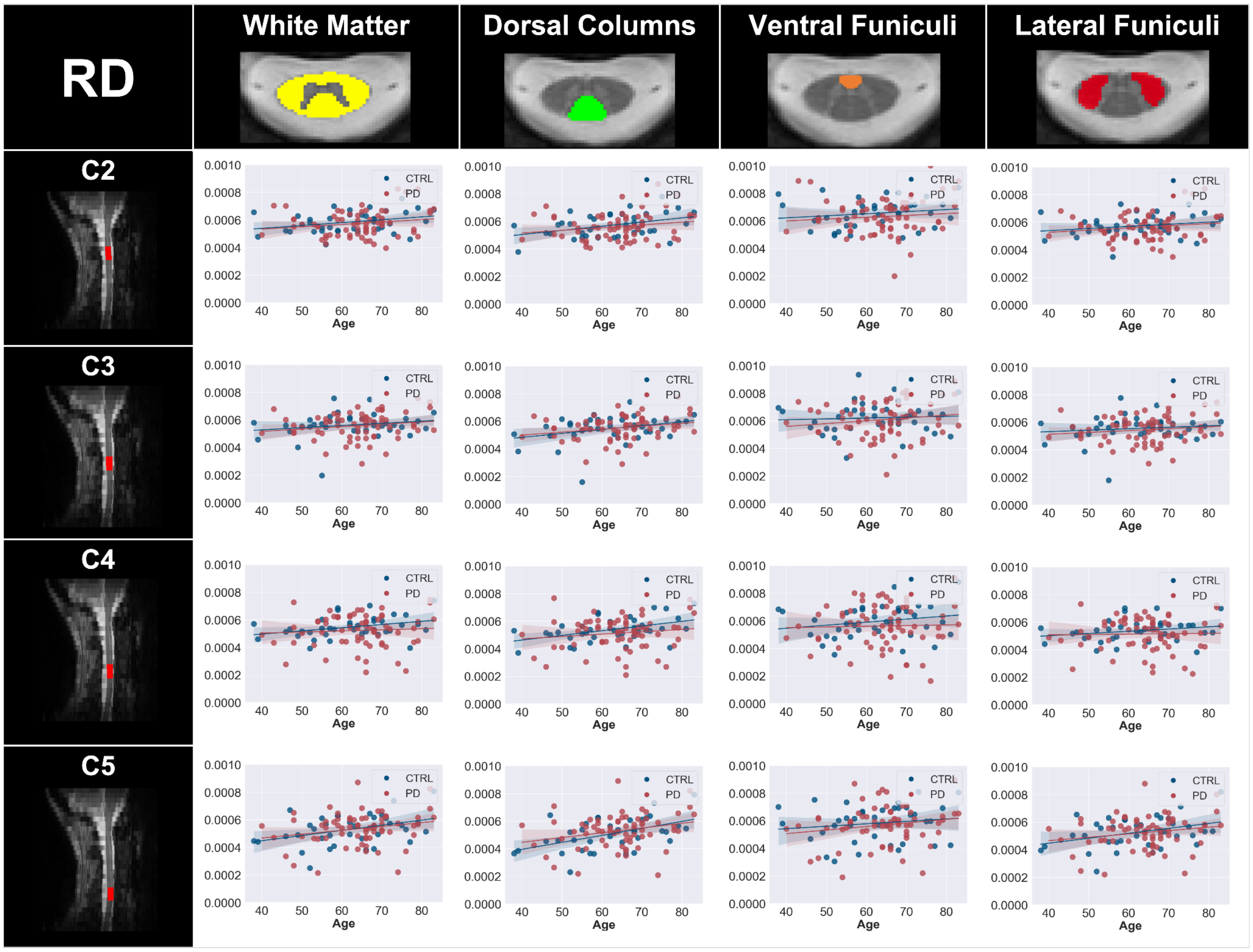

Figure II-4. RD values with respect to age for healthy controls (CTRL) and PD subjects across spinal levels C2 to C5, for the entire white matter and subregions: dorsal columns, ventral funiculi, and lateral funiculi.

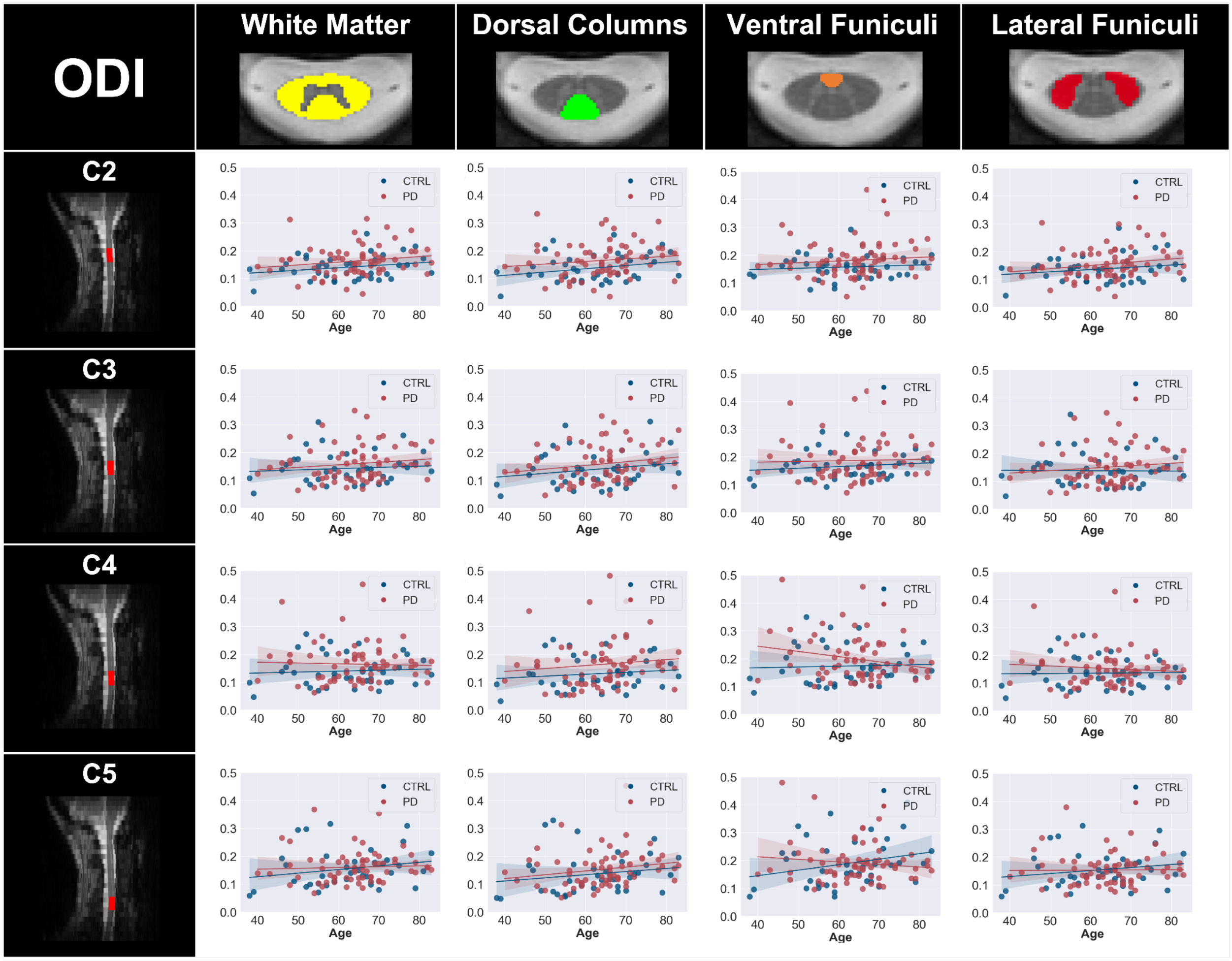

Figure II-5. ODI values with respect to age for healthy controls (CTRL) and PD subjects across spinal levels C2 to C5, for the entire white matter and subregions: dorsal columns, ventral funiculi, and lateral funiculi.

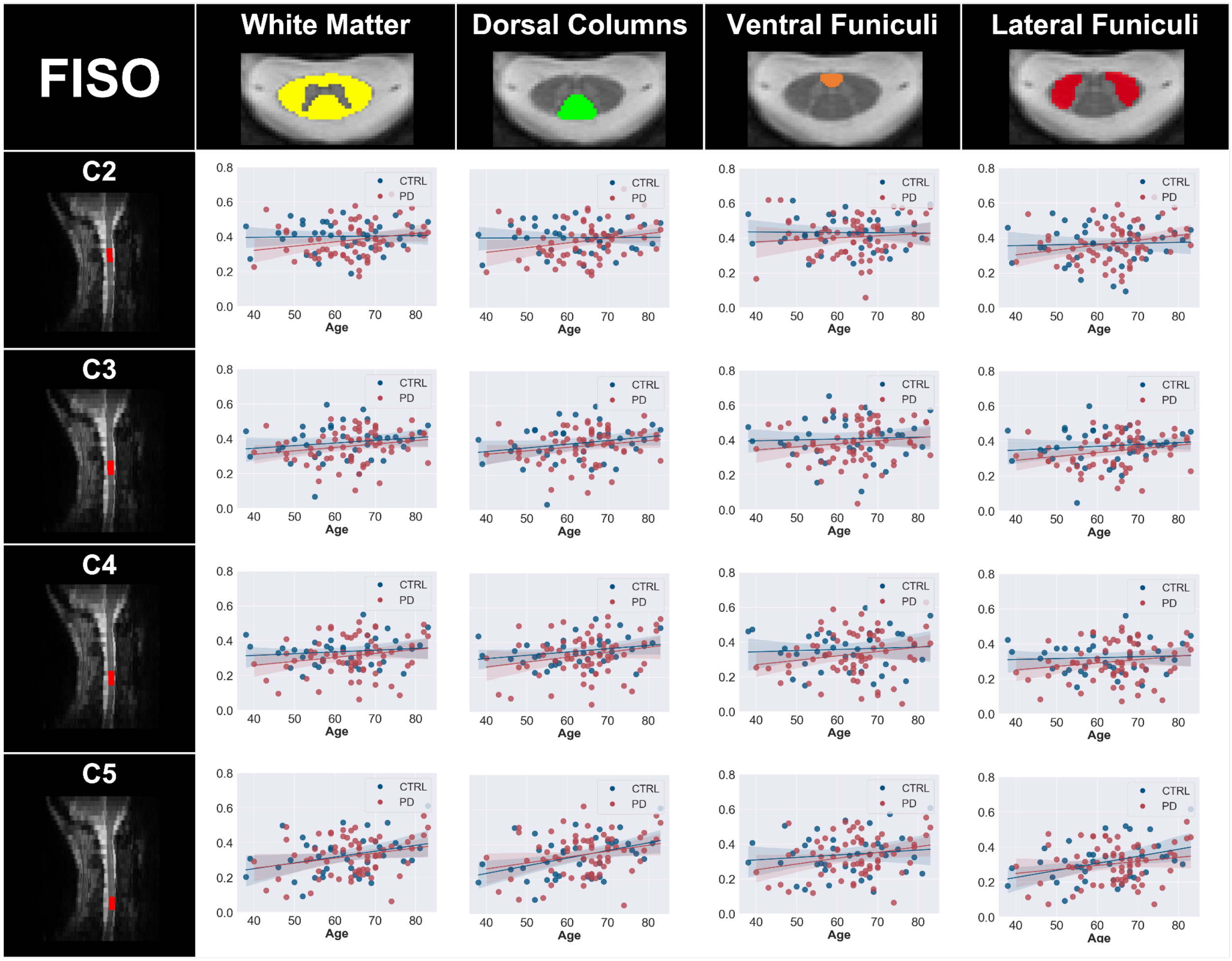

Figure II-6. FISO values with respect to age for healthy controls (CTRL) and PD subjects across spinal levels C2 to C5, for the entire white matter and subregions: dorsal columns, ventral funiculi, and lateral funiculi.

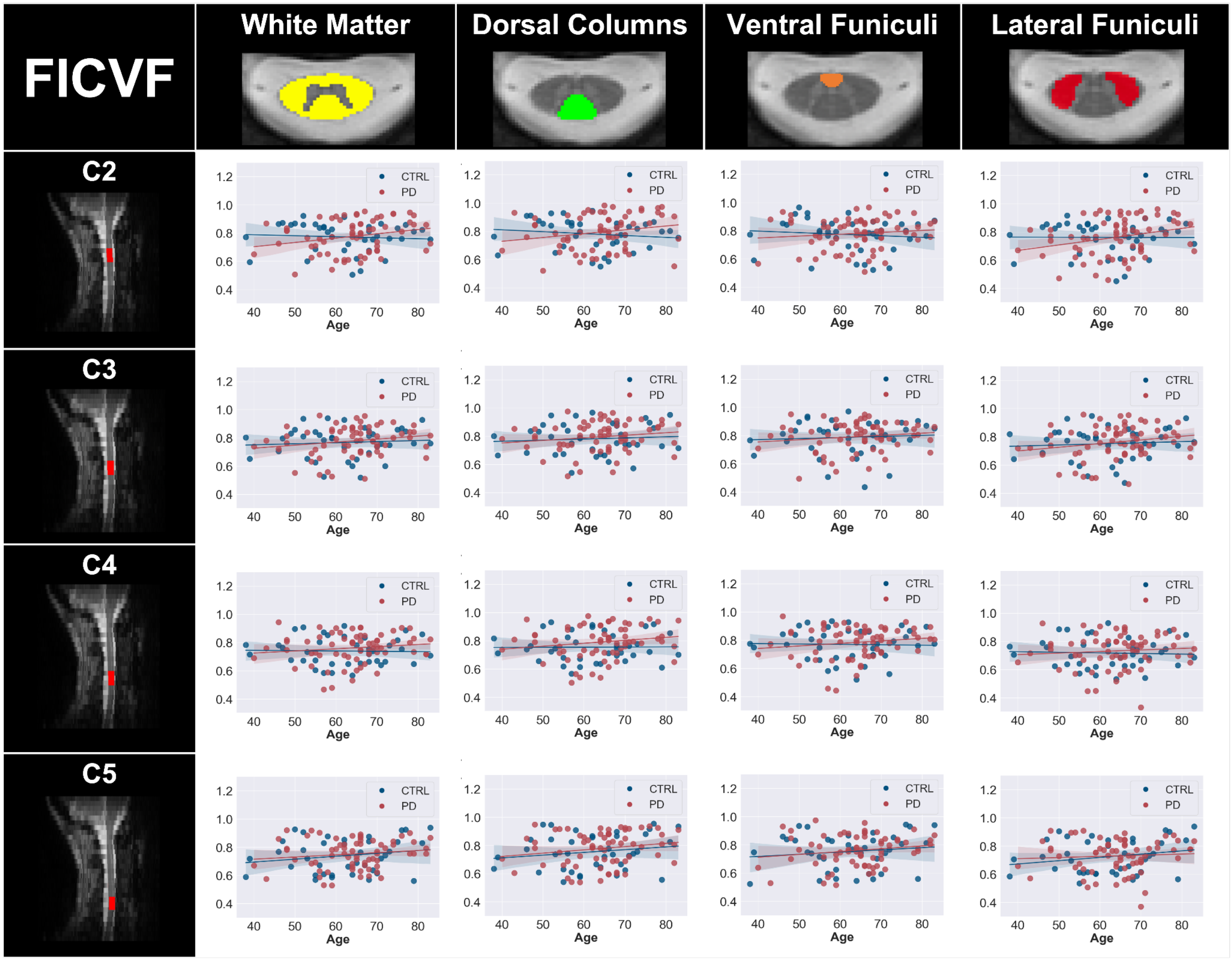

Figure II-7. FICVF values with respect to age for healthy controls (CTRL) and PD subjects across spinal levels C2 to C5, for the entire white matter and subregions: dorsal columns, ventral funiculi, and lateral funiculi.

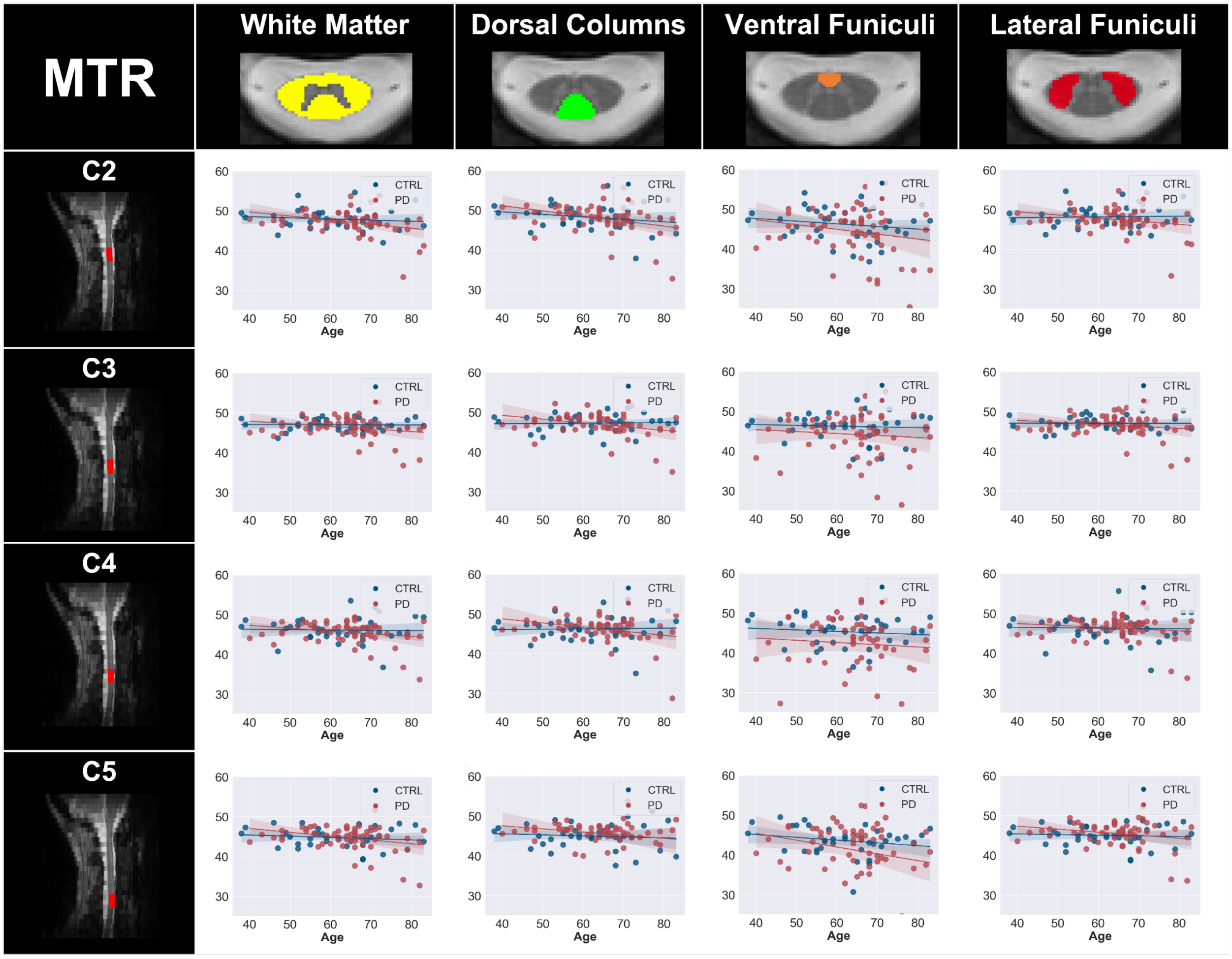

Figure II-8. MTR values with respect to age for healthy controls (CTRL) and PD subjects across spinal levels C2 to C5, for the entire white matter and subregions: dorsal columns, ventral funiculi, and lateral funiculi.

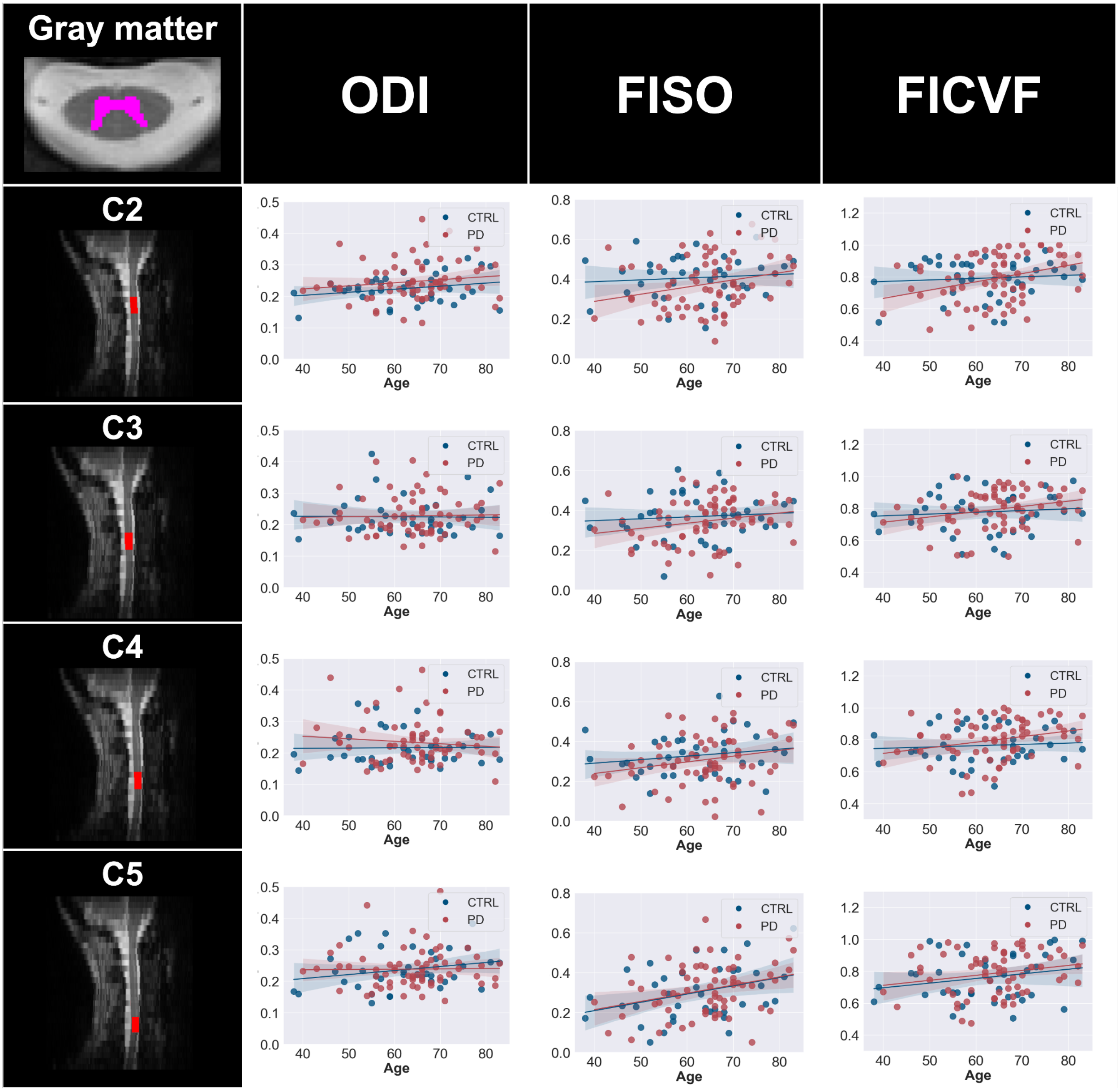

Figure II-9. NODDI (ODI, FISO and FICVF) values with respect to age for healthy controls (CTRL) and PD subjects across spinal levels C2 to C5, for the entire gray matter.

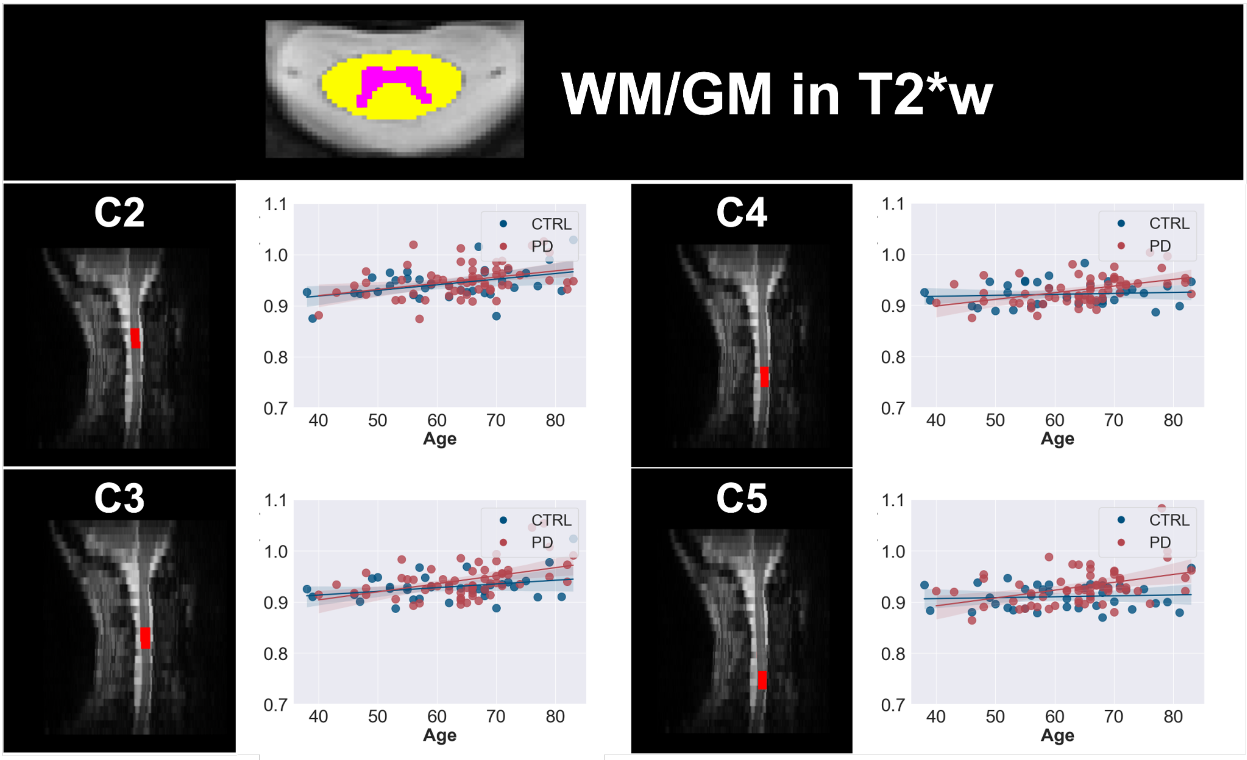

Figure II-10. WM/GM ratio in T2*w with respect to age for healthy controls (CTRL) and PD subjects across spinal levels C2 to C5.

Appendix C: Correlations of CSA with the UPDRSIII score and with age

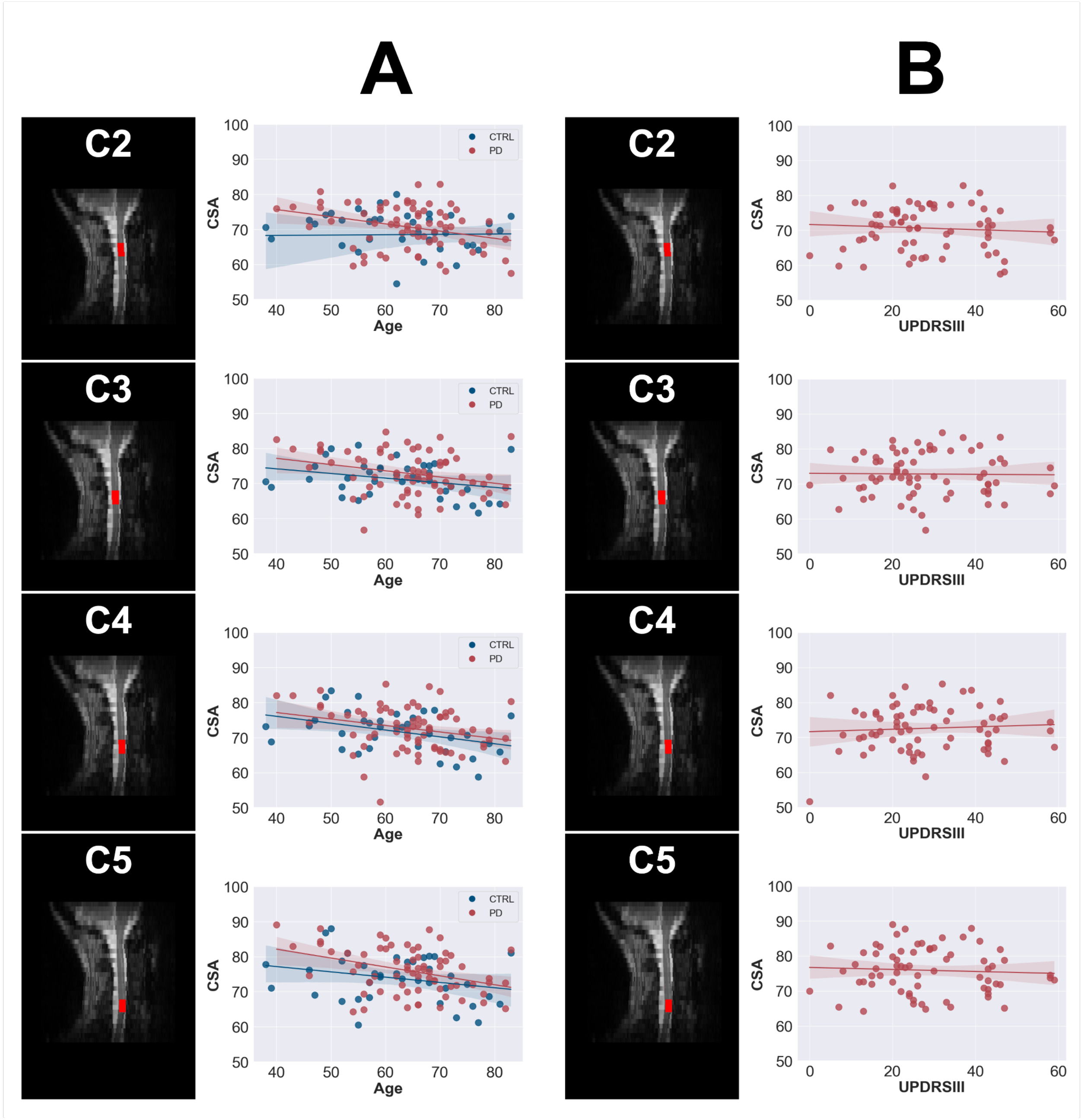

Figure III-1: Spinal cord cross-sectional area (CSA) in correlation to Age (A) and the UPDRSIII score (B) across spinal levels C2 to C5.
